# Multiple Roles of G3BP1 in Regulating STING-Dependent Interferon and Cytokine Induction by Cytosolic dsDNA and HSV-1 Infection

**DOI:** 10.3390/v18070719

**Published:** 2026-06-30

**Authors:** Trupti Devale, Praveen Manivannan, Krishnamurthy Malathi

**Affiliations:** 1 Department of Molecular, Cellular and Developmental Biology, University of Toledo, 2801 West Bancroft Street, Toledo, OH 43606, USA; trupti.devale@rockets.utoledo.edu (T.D.); manivann@umich.edu (P.M.); 2 Department of Microbiology and Immunology, University of Michigan Medical School, Ann Arbor, MI 48109, USA

**Keywords:** G3BP1, STING, cGAS, HSV-1, 2′3′cGAMP, stress granules, PERK, innate immunity

## Abstract

Virus infection requires coordinated activation of pathogen-sensing, innate immune, and cellular stress response pathways to mount an effective antiviral defense. Recognition of nucleic acid pathogen-associated molecular patterns (PAMPs) by pattern recognition receptors (PRRs) initiates signaling cascades that drive the production of type I interferons (IFNs) and proinflammatory cytokines. These responses are often accompanied by the activation of integrated stress response pathways that help optimize host defense. Cytosolic double-stranded dsDNA, generated during viral infection or released from damaged mitochondria, is sensed by cyclic GMP-AMP synthase (cGAS), which generates 2′3′-cGAMP to activate stimulator of interferon genes (STING). Activated STING translocates from the endoplasmic reticulum to the Golgi, where it drives TBK1-dependent IFN and cytokine production. Previous reports show that cGAS activity is enhanced by Ras-GAP SH3 domain binding protein 1 (G3BP1), a key nucleator of stress granules (SGs), independent of its role in SG assembly. Here, we identify a non-canonical role of G3BP1 as a regulator of DNA sensing responses at multiple levels, including STING intracellular trafficking, in addition to potentiating cGAS activity. Loss of G3BP1 impaired STING-dependent IFN and cytokine responses to HSV-1 infection and viral DNA. G3BP1-deficient cells showed reduced cGAMP-induced STING translocation to the Golgi, induction of type I IFN and proinflammatory cytokines, and activation of the ER stress kinase PERK and stress granule formation. Together, these findings demonstrate G3BP1-STING as a node linking DNA sensing, innate immunity, and stress signaling with broad implications for antiviral defense and diseases characterized by aberrant DNA sensing and stress responses, including neurodegeneration, fibrosis, and autoimmunity.

## 1. Introduction

During viral infections, specialized cytosolic pattern recognition receptors (PRRs) such as retinoic acid-inducible gene I (RIG-I)-like receptor (RLR), cytosolic DNA receptor (CDR) and endosomal Toll-like Receptor (TLR) families of proteins detect viral RNA and DNA genomes and replication intermediates as pathogen-associated molecular patterns (PAMPs), and induce signaling pathways that promote the expression and secretion of IFN and cytokines [1,2]. Cytosolic dsDNA, pathogenic or of endogenous origin, is detected in most cell types by the primary sensor cyclic GMP–AMP synthase (cGAS) and activated cGAS catalyzes the synthesis of cyclic GMP-AMP (2′3′cGAMP, cGAMP) from ATP and GTP, which binds with and activates stimulator of IFN genes (STING) on the endoplasmic reticulum (ER) membrane [3,4,5,6,7,8]. Under resting conditions, STING assumes a dimeric structure with the C-terminal ligand-binding domain facing the cytoplasm [9]. Upon binding 2′3′cGAMP, STING undergoes conformational change that results in its oligomerization and robust trafficking from the ER to the Golgi region, where Tank-binding kinase 1 (TBK1) is recruited to activate both Interferon regulatory factor 3 (IRF3) and nuclear factor-κB (NF-κB) transcription factors that upregulate the expression of type I interferons (IFNs) and inflammatory cytokines [10,11,12,13,14,15,16,17]. STING translocation from the ER to Golgi is essential for the immune response and this pathway is regulated at many levels, involving post-translational modifications on STING by phosphorylation, palmitoylation, ubiquitination and SUMOylation that affect STING activity, stability or trafficking [18,19,20,21,22,23]. Multiple proteins, through direct interaction or indirectly, function to retain STING in the ER, facilitate transport to the Golgi, or redirect to endolysosomes for degradation to terminate signaling, which can strongly reshape both antiviral and non-canonical STING functions, adding extra layers of regulation of this pathway [24,25,26,27].

STING exerts IFN-independent non-canonical functions that extend beyond innate immune signaling. STING acts as a proton channel inducing non-canonical autophagy by LC3-lipidation on single membrane vesicles that regulates endolysosomal homeostasis and cell death [28,29,30,31]. In response to Ionizing Radiation-induced DNA damage, STING activates non-canonical NF-κB signaling by NF-κB-inducing kinase (NIK) to suppress IFNβ production in dendritic cells [32,33]. On 2′3′cGAMP binding, STING binds and directly activates ER stress kinase Protein kinase RNA-like ER kinase (PERK) that phosphorylates eIF2α to control protein translation in cellular senescence and organ fibrosis [34]. We showed in recent studies that PERK activated by DNA damage agents and immunostimulatory DNA induced the formation of SG and the STING-PERK-G3BP1 axis-coordinated stress response and innate signaling [35]. A recent study showed that STING binds with the stress granule marker, G3BP1 (Ras-GAP SH3 domain binding protein 1), and functions as a scaffold for SG before the condensation and maturation at the ER [36].

In virus-infected cells, activation of innate immune signaling is often accompanied by integrated stress response pathways to optimize the host response to clear infections. Accumulation of viral nucleic acids in the cytosol and of viral proteins at the ER triggers stress kinases such as protein kinase R (PKR) and PERK to initiate the integrated stress response (ISR) by phosphorylating the alpha subunit of the eukaryotic translation initiation factor-2 (eIF2α), resulting in translation shut-off and the formation of SG [37,38,39]. G3BP1 is a core SG protein, and the intrinsic-disordered regions in the protein initiate multivalent RNA–protein interactions to establish liquid–liquid phase separation (LLPS) from the rest of the cytoplasm, facilitating the recruitment of multiple proteins into a dynamic compartment [39,40,41,42]. Recent studies highlight antiviral SG (avSG) assembly as a signaling platform to connect the stress sensors and innate immune effectors to mount the antiviral response by enhancing type I interferon (IFN) production [43,44,45,46,47]. We showed recently that DNA damage agents activate PERK via STING and induce SG formation, and cells lacking G3BP1 showed reduced STING-induced signaling and cytokine production [35].

In a non-canonical role, G3BP1 promotes cGAS-mediated IFN production by serving as a co-factor to enhance cGAS DNA sensing, by increasing the affinity of cGAS for DNA and promoting cGAS oligomerization and LLPS [48,49]. However, whether G3BP1 regulates STING beyond 2′3′cGAMP production, particularly at the ER, remains unclear. In this study, we show that G3BP1 exerts its regulation of the cGAS-STING pathway at multiple levels. In addition to supporting responses to HSV-1 infection and HSV60 viral DNA, G3BP1 participates in optimal STING activation after cGAMP stimulation, indicating a role downstream of cGAS. Mechanistically, G3BP1 facilitates 2′3′cGAMP-induced STING aggregation, and transit from the ER to Golgi, as cells lacking G3BP1 or expressing mutated forms of G3BP1 which are functionally defective showed reduced aggregation and continued ER retention in response to 2′3′cGAMP, which bypasses cGAS. Loss of G3BP1 impaired STING-dependent IFN and cytokine induction and ER stress responses to HSV-1 infection and viral DNA. Together, these findings identify G3BP1 as a regulator of STING trafficking and STING-mediated stress signaling during DNA virus infection.

## 2. Materials and Methods

### 2.1. Chemicals, Reagents, Plasmids, and Antibodies

Chemicals, unless otherwise indicated, were from Sigma-Aldrich (St. Louis, MO, USA). STING ligands poly dA:dT, HT-DNA, HSV60 or 2′3′cGAMP, and cGAS inhibitor G140 were from InvivoGen (San Diego, CA, USA). PERK inhibitor GSK2656157 was from Santa Cruz Biotechnology (Dallas, TX, USA), and Epigallocatechin Gallate (EGCG, 70935) and cycloheximide (14126) were purchased from Cayman Chemicals (Ann Arbor, MI, USA). Poly I:C was from Calbiochem (San Diego, CA, USA). The HA-STING plasmid was from Saurabh Chattopadhyay, University of Kentucky, pEGFP-C1-G3BP1-WT was a gift from Anthony Leung (Addgene plasmid # 135997; http://n2t.net/addgene:135997, accessed on 11 May 2025; RRID:Addgene_135997), pMSCV-hygro-STING (Addgene 102598), pMSCV-hygro-STING HAQ (Addgene 102600), pMSCV-hygro-STING HAQ+V155M (Addgene 102601), and pMSCV-hygro-STING V155M were a gift from Nicolas Manel (Addgene plasmid # 102599; http://n2t.net/addgene:102599, accessed on 11 May 2025; RRID:Addgene_102599), and STING-V1 and STING-V2 were a gift from Eric Schirmer (Addgene plasmid # 124262; http://n2t.net/addgene:124262, accessed on 11 May 2025; RRID:Addgene_124262, Addgene_124264). GFP-G3BP1 F33W, K376Q, and F380/382L were constructed by site-directed mutagenesis using the primers listed in Table 1 and the QuikChange Lightning Multi Site-Directed Mutagenesis Kit (Agilent Technologies, Santa Clara, CA, USA) and confirmed by sequencing. Antibodies to p-STING (50907), STING (13647), p-TBK1 (5482), TBK1 (3013), p-IRF3 (4947), IRF3 (4302), p-STAT1 (9167), STAT1 (9172), p-eIF2α (3398), eIF2α (5324), β-actin (3700), and PERK (3192), and anti-mouse IgG and anti-rabbit IgG horseradish peroxidase (HRP)-linked secondary antibodies, were from Cell Signaling Technology (Danvers, MA, USA), G3BP1 (sc-81940), golgin 97 Antibody (CDFX): sc-59820, and TIA1 (sc-166247) were from Santa Cruz Biotechnology (Dallas, TX, USA), cGAS (26416-1-AP) and BiP (66574-1-Ig) were from Proteintech (Rosemont, IL, USA), and p-PERK (MA5-15033) and puromycin (MABE343) were from Invitrogen, Life Technologies, Carlsbad, CA, USA). Enhanced chemiluminescence (ECL) reagents were from Boston Bioproducts (Ashland, MA, USA) and Super-Signal West Pico Chemiluminescent substrate (Pierce Chemical, Rockford, IL, USA).

### 2.2. Cell Culture, Stable Cell Lines, and Transfections

HT1080 (a gift from Ganes Sen, Cleveland Clinic, Cleveland, OH, USA), G3BP1 KO [43], STING KO [35], WT and *PERK*^−/−^ mouse embryonic fibroblasts (MEFs, a gift from Maria Hatzoglou, Case Western Reserve University, Cleveland, OH, USA), NuFF (human newborn foreskin fibroblast cells, a gift from Saurabh Chattopadhyay, University of Kentucky), Phoenix (a gift from Fan Dong, University of Toledo), Vero (gift from Travis Taylor, University of Toledo), and HT1080 stably expressing GFP-G3BP1 [35] were cultured in Dulbecco’s modified minimal essential medium with 10% fetal bovine serum and 100 μg/mL penicillin/streptomycin in a 5% CO_2_ incubator at 37 °C. G3BP1 KO stable cells expressing WT or G3BP1 mutants were selected in the presence of 500 μg/mL neomycin and clones expressing similar protein levels were expanded. For stable expression of STING WT and mutant proteins in STING KO cells, retroviral constructs were packaged in Phoenix cells, transduced into target cells and selected in 200 μg/mL hygromycin and expanded. Knockout cells for TIA1 in HT1080 cells were generated using the CRISPR/Cas9 system and a guide RNA targeting exon 3 of TIA1 (5′-GCAGCATTAGCTGCTATGAA-3′). Clones were generated by limiting dilution, and gene knockout clones were validated by immunoblotting and expanded. Plasmids, STING ligands poly dA:dT (5 μg/mL), HT-DNA (4 μg/mL), HSV60 (5 μg/mL), 2′3′cGAMP (5 μg/mL) or polyI:C (2 μg/mL) were transfected using lipofectamine 2000 (Invitrogen, Carlsbad, CA, USA), according to the manufacturer’s protocol. Mock controls included cells treated with the transfection reagent alone. In experiments involving inhibitors, cells were preincubated with inhibitor GSK2656157 (5 μM) or vehicle for mock control for 1h prior to transfection and then replaced with growth medium.

### 2.3. Virus Infection and Plaque Assays

HSV-1 (KOS, provided by Saurabh Chattopadhyay, University of Kentucky) at a multiplicity of infection of 1 was used to infect target cells in serum-free media, and after 1 h, replaced with growth medium. HSV-1 stock was UV-inactivated by exposure twice to 254 nM UV at 600 mJ/cm^2^ × 100 in a Stratalinker (Stratagene, Agilent technologies, Santa Clara, CA, USA). The effectiveness of UV-inactivation was confirmed by determining the viral growth and titer in plaque assays. For viral titers, Vero cells were incubated for 1 h with 10-fold serial dilutions of supernatants collected after infection, overlaid with DMEM containing 1.5% carboxymethylcellulose and 2% FBS and incubated for 72 h. The cells were fixed with methanol and stained using 0.1% crystal violet and plaques were counted. The assays were performed in triplicate and fold change in virus titers were determined from three independent experiments.

### 2.4. RNA Isolation and Quantitative Real-Time PCR

Total RNA was isolated from cells using TRIzol reagent (Invitrogen), as per the manufacturer instructions. The cDNAs were synthesized with random decamers and a RETROscript cDNA synthesis kit (Life Technologies; Thermo Fisher Scientific, Carlsbad, CA, USA). Real-time quantitative PCR (qRT-PCR) was performed using SYBR Green PCR Master Mix (Bio-Rad Laboratories Inc., Hercules, CA, USA) and target gene-specific primers (Table 2), target amounts were measured as cycle threshold (CT) values, and the results were analyzed by the ΔΔCt method. Target gene mRNA expression was normalized to *GAPDH* expression. The analysis was performed from three or more independent biological experiments and represented as mean ± SD.

### 2.5. Split Venus (BiFC) Assay for STING Dimerization and Activity

STING-V1 (N-terminus of Venus) and STING-V2 (C-terminus of Venus) plasmids were transfected into HT1080 WT or G3BP1 KO cells, and 24 h later, transfected with synthetic dsDNA, pdA:dT (5 μg/mL) or 2′3′cGAMP (5 μg/mL), for the indicated times to monitor dimerization in live cells by measuring fluorescence using the EVOS M5000 fluorescence microscope (Thermo Scientific, Life Technologies, Carlsbad, CA, USA). Control cells transfected with single plasmids and transfected with both plasmids and transfection reagent alone were used to normalize. The % cells showing fluorescence from at least 3 random fields from a minimum of 100 cells from three independent wells was plotted as mean ± SD.

### 2.6. Puromycin Incorporation Assay and OP-Puro Labeling and Detection by Fluorescence Microscopy

Cells were plated in 6-well plates and transfected with HA-STING plasmid (for 24 h) or with STING ligands HT-DNA (4 μg/mL), HSV60 (5 μg/mL) or 2′3′cGAMP (5 μg/mL) for 6 h. Puromycin dihydrochloride (Sigma-Aldrich, Cat# P8833) was then added to the culture medium at a final concentration of 10 µg/mL, and cells were incubated for 30 min at 37 °C to label newly synthesized proteins. Control samples were mock-transfected or treated with cycloheximide (50 µg/mL) for 20 min followed by puromycin treatment. Cells were lysed in NP40 lysis buffer, and whole-cell lysates were subjected to Western blot analysis. Nascent proteins labeled with puromycin were detected using anti-puromycin antibody (1:8000) and normalized with β-actin used as a loading control. Densitometric analysis was performed using ImageJ to quantify the incorporation of puromycin, and the values were normalized to control conditions and β-actin and plotted. For imaging protein synthesis, cells were seeded on glass coverslips and treated with HSV60 (5 μg/mL) or 2′3′cGAMP (5 μg/mL) for 6 h or infected with HSV-1 or UV-inactivated HSV-1 at MOI = 1.0 for 24 h. Cells were treated in parallel with cycloheximide (CHX), a well-established translation inhibitor, as a positive control for translational inhibition. Protein synthesis was detected in cells using fluorescence microscopy and a Click-iT OPP protein synthesis assay kit (Invitrogen, Thermo Fisher Scientific, Waltham, MA, USA), following the manufacturer’s instructions. At indicated time points, Click-iT OPP reagent (20 mM) was added to cells for 30 min and cells were fixed with 4% paraformaldehyde (Boston Bioproducts, Ashland, MA, USA) for 15 min at room temperature and then permeabilized with 0.5% Triton X-100 in PBS for 15 min at room temperature. Freshly made Click-iT Plus reaction cocktail was added to cells for 30 min at room temperature. Cells were then blocked with 3% BSA, 0.02% Tween in PBS for 1 h at room temperature and then incubated for 2 h with anti-G3BP1 antibody (1:1000) diluted in 3% BSA. Cells were then incubated with a secondary antibody (Alexa 647-conjugated anti-immunoglobulin antibody, Molecular Probes; Eugene, OR, USA) for 1 h at room temperature, washed with PBS and mounted in Fluoro-Gel II (EMS catalog #17985-50) containing DAPI (4′,6-diamidino-2-phenylindole) to counterstain the nucleus. Fluorescence images were captured using an Olympus IX81 Microscope (Olympus Corporation, Tokyo, Japan). Random images were captured and further analysis and processing of images were performed using ImageJ software (v1.5) or Olympus Stream View software V2.3 (Olympus Corporation, Tokyo, Japan). Incorporation of OPP as an indicator of protein translation was compared to cells forming SG puncta and compared to mock-treated cells for 2′3′ cGAMP and HSV60 and uninfected and UV-inactivated HSV-1 for HSV-1-infected cells. The percentages of SG-containing cells (red) and showing reduced OPP incorporation (green) are indicated by arrows. The %SG positive cells in HSV-1-infected cells were calculated in at least five random fields from a minimum of 100 cells and plotted as mean ± SD.

### 2.7. Quantitation of Stress Granules in Live Cells

Cells stably expressing GFP-G3BP1 (WT or mutants) were seeded in triplicates in 12-well plates and transfected with STING ligands or infected with HSV-1 as indicated. Inhibitors were added at indicated concentrations 1 h prior to transfection or infection and added post transfection or viral adsorption. Mock-transfected cells not receiving DNA ligands or cells treated with vehicles used for inhibitors or UV-inactivated HSV-1 were used as controls for non-specific stress effects. Formation of stress granules (G3BP1 puncta) was monitored using an EVOS M5000 fluorescence microscope (Thermo Scientific, LifeTechnologies, Carlsbad, CA, USA) at the indicated times. For stress granule analysis, cells containing G3BP1 puncta (*n* > 5), characteristic of SGs, and that were above 0.6 μm in diameter were considered as previously [50]. The percentages of GFP-positive cells forming SGs were calculated in at least five random fields from a minimum of 100 cells per treatment from three independent experiments and plotted as mean ± SD.

### 2.8. Immunoblotting

Immunoblot analysis was performed using previously described procedures [35,43]. Briefly, cells treated as described were lysed with Nonidet P-40 lysis buffer (0.5% NP-40, 90 mM KCl, 5 mM magnesium acetate, 20 mM Tris, pH 7.5, 5 mM β-mercaptoethanol, 0.1M phenylmethylsulfonyl fluoride (PMSF), 0.2 mM sodium orthovanadate, 50 mM NaF, and 10 mM glycerophosphate), supplemented with protease inhibitor mixture (Roche Diagnostics GmbH, Mannheim, Germany). Total protein extracts were separated by SDS-PAGE, transferred to nitrocellulose membranes (Bio-Rad, Hercules, CA, USA), and probed with the indicated antibodies. Immunoreactive bands were visualized using chemiluminescent reagents from Boston Bioproducts (Ashland, MA, USA) or Super-Signal West Pico Chemiluminescent substrate (Pierce Chemical, Rockford, IL, USA) and a ChemiDoc-It2 510 imager (UVP, Fisher Scientific; Hampton, NH, USA). The results shown are representative of at least two experiments or as indicated in legends. Images were processed using Adobe Photoshop CC v20.0.5 (Adobe, San Jose, CA, USA). In some instances, non-specific lanes were cropped to generate the images, and the boundaries are indicated as shown. The Western blot band density on immunoblots was quantified using ImageJ software (version 1.53t; National Institutes of Health, USA).

### 2.9. Immunofluorescence Assays

Cells were grown on glass coverslips, and after the indicated treatments, the cells were fixed in 4% paraformaldehyde (Boston Bioproducts, Ashland, MA, USA) in phosphate-buffered saline (PBS) for 15 min at room temperature. Cells were then permeabilized with 0.2% Triton X-100 in phosphate-buffered saline (PBS) for 15 min and blocked with 3% bovine serum albumin (BSA) and 0.02% Tween 20 in PBS (blocking buffer) for 1 h at room temperature. Cells were then washed with PBS and incubated overnight at 4 °C with G3BP1 antibodies (1:250) in blocking buffer. For cells stained with STING, BiP and Golgin97 cells were fixed in 100% pre-chilled methanol (Fisher Scientific) for 30 min at −20 °C, permeabilized with 0.5% Triton X-100 in phosphate-buffered saline (PBS) for 15 min, and blocked with 5% fetal bovine serum (FBS) and 0.2%Tween 20 for 1 h at room temperature. Cells were then washed with PBS and incubated overnight at 4 °C with the indicated antibodies (STING—1:600, BiP—1:500, and Golgin97—1:250) diluted in 5% fetal bovine serum (FBS) and 0.2%Tween in PBS. The same buffer was further used for the dilution of secondary antibodies in the following cell-staining steps. Unbound primary antibodies were washed with PBS, and cells were further stained with Alexa 488- or Alexa 647-conjugated anti-immunoglobulin antibody (1:1500) (Molecular Probes, Eugene, OR, USA) secondary antibody. Cells were then washed more than three times to remove any unbound secondary antibodies and mounted in Fluoro-Gel II (EMS catalog #17985-50) containing DAPI (4′,6-diamidino-2-phenylindole) to counterstain the nucleus. Positive and negative controls were included to rule out nonspecific staining and any cross-talks between channels. Fluorescence images were captured using a Leica CS SP5 multiphoton laser-scanning confocal microscope (Leica Microsystems, Wetzlar, Germany) or Olympus IX81 Microscope (Olympus Corporation, Tokyo, Japan). Random images were captured and further analysis and processing of images were performed using ImageJ software (v1.5) or Olympus Stream View software V2.3 (Olympus Corporation, Tokyo, Japan). Colocalization of proteins was assessed by line scan analysis and the intensities along the line were measured using a plot profile using ImageJ software (version 1.53t; National Institutes of Health, USA). For stress granule analysis, cells containing G3BP1 puncta (*n* > 5), characteristic of SGs, and that were above 0.6 μm in diameter were considered as previously described [43,50]. The percentages of SG-containing cells were calculated in at least five random fields from a minimum of 100 cells per treatment from three independent experiments and plotted as mean ± SD.

### 2.10. Quantification and Statistical Analysis

All data were statistically analyzed using GraphPad Prism software (V8, Boston, MA, USA) and Python 3.12. Unpaired Welch’s *t*-tests were used for comparing two datasets as indicated. One-way ANOVA followed by Dunnett’s multiple comparisons test was used for comparisons among three or more groups to a control. Two-way ANOVA with Holm–Sidak-corrected planned comparisons was used for time-course or two-factor experiments. For all analyses, a *p*-value of 0.05 or below was considered statistically significant. Error bars in all data represent standard deviations, unless otherwise stated as the standard error of the mean. Results shown from Western analyses and confocal experiments are representative of at least two or more independent experiments. The band density was quantitated for the individual lanes for the Western blot we have shown in each figure. All assays were performed in at least two separate biological replicates, with each replicate comprising a minimum of three technical repeats. The exact numbers of biological and technical repeats are detailed in the corresponding figure legends.

## 3. Results

### 3.1. G3BP1 Is Required for DNA Virus or Viral DNA-Induced Innate Immune Response

**Figure 1:**
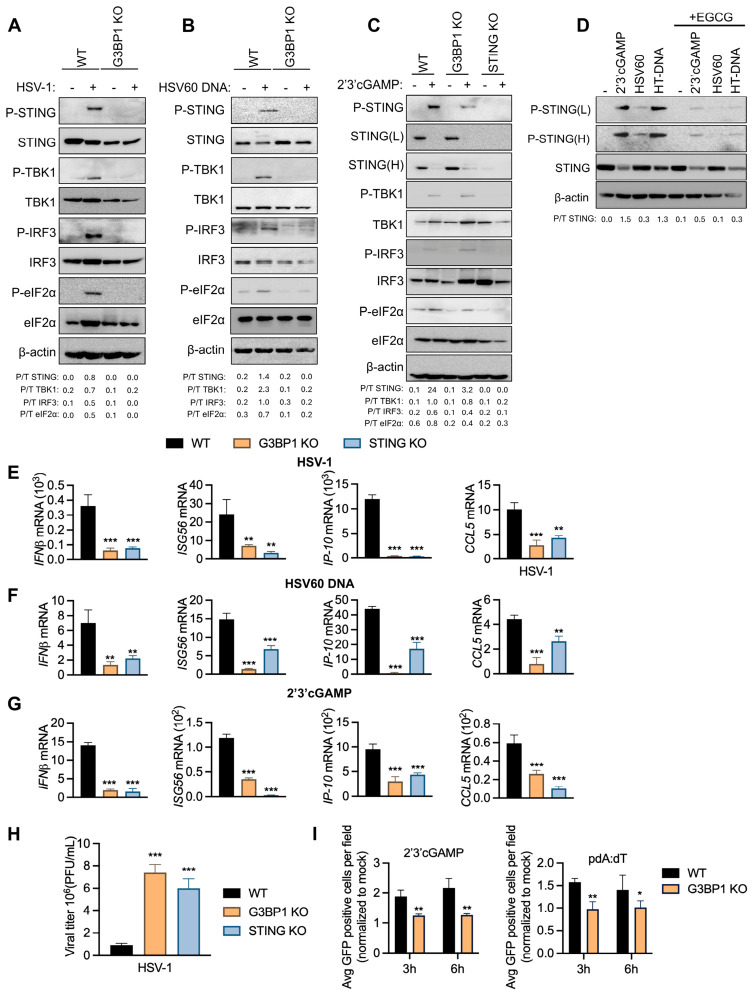
G3BP1 regulates innate immune signaling by the DNA virus and viral DNA. HT1080 WT and G3BP1 KO cells were (**A**) infected with HSV−1 (MOI = 1.0) or (**B**) transfected with HSV−60 (5 μg/mL) for 6 h and cell lysates were analyzed for phosphorylation of STING, TBK1, IRF3 and eIF2α and compared to total protein levels and loading, normalized to β-actin levels. The ratios of the indicated phospho-proteins/total proteins for the antibodies probed were quantified by ImageJ. (**C**) HT1080 WT, G3BP1 KO and STING KO cells were transfected with 2′3′−cGAMP (5 μg/mL) for 6 h and cell lysates were probed with the indicated phospho- and total antibodies on immunoblots; two exposures of STING (L, low and H, high) of the same blot are shown. The ratios of the indicated phospho-proteins/total proteins for the antibodies probed were quantified by ImageJ. (**D**) HT1080 cells were transfected with 2′3′-cGAMP (5 μg/mL), HSV60 (5 µg/mL), or HT−DNA (4 µg/mL) without or with the G3BP1 inhibitor, EGCG (20 μM), for 6 h and the phosphorylation of STING was determined in immunoblots, compared to total STING and normalized to β-actin. The ratios of pSTING/total STING were quantified by ImageJ. HT1080 WT, G3BP1 KO and STING KO cells were (**E**) infected with HSV−1 (MOI = 1.0) for 24 h, (**F**) transfected with HSV−60 (5 μg/mL) for 6 h, and (**G**) transfected with 2′3′−cGAMP (5 μg/mL) for 6 h and mRNA levels of *IFNβ*, *ISG5*6, *IP-10* and *CCL5* were measured by qRT-PCR and normalized to *GAPDH* mRNA levels and plotted as fold induction. Data represents the mean ± SE from three independent experiments. *p* values in (**E**–**G**) were calculated by one-way ANOVA with Dunnett’s multiple-comparisons test compared to WT cells. (**H**) HT1080 WT, G3BP1 KO and STING KO cells were infected with HSV−1 (MOI = 1.0) for 24 h and the virus titer in supernatants was determined by plaque assay. Data shown are from experiments performed in triplicate and at least repeated twice; the graph shows the mean ± SD, *n* = 3. *p* values were calculated by one-way ANOVA with Dunnett’s multiple-comparisons test compared to the titer in WT cells. (**I**) STING−V1 and STING−V2 Venus fusion plasmids were transfected into HT1080 WT or G3BP1 KO cells, and 24 h later, transfected with 2′3′−cGAMP (5 μg/mL) or synthetic dsDNA, pdA:dT (5 μg/mL), and at the indicated times, the reconstituted fluorescence was measured in live cells using an EVOS M5000 fluorescence microscope. Control cells transfected with single plasmids, transfected with both plasmids, and with transfection reagent alone were used to normalize. The average % cells showing fluorescence from at least three random fields from a minimum of 100 cells from three independent wells was plotted as the mean ± SD. *p* values were calculated by two-way ANOVA with Holm–Sidak-corrected planned comparisons comparing G3BP1 KO to WT cells at each time point. The results presented here are representative of at least three biological repeats or as indicated. * *p* < 0.05; ** *p* < 0.01; *** *p* < 0.001.

To investigate the role of G3BP1 in the cellular response to the DNA virus and virus-derived DNA that may act as a PAMP, we used HSV-1 to infect cells and HSV-60, a 60-base pair, double-stranded (ds) DNA oligonucleotide containing viral DNA motifs derived from the HSV-1 genome, to transfect cells. HT1080 WT and G3BP1 KO cells were infected with HSV-1 (MOI = 1) for 24 h or transfected with HSV-60 (5 μg/mL) for 6 h, and the activation of the STING-mediated innate immune response was analyzed in cell lysates. WT cells, after HSV-1 infection or HSV-60 transfection, showed phosphorylation of STING (pSTING), an indicator of activation, that induced downstream signaling by phosphorylation of TBK1 (pTBK1) and IRF3 (pIRF3). We recently showed that diverse cytosolic dsDNA ligands activated cGAS/STING signaling and the ER stress pathway, leading to phosphorylation of eIF2α (peIF2α) and stress granule formation [35]. Infection with HSV-1 or viral DNA HSV-60 resulted in the induction of ER stress as shown by the increase in peIF2α compared to control cells. G3BP1 is required for enhancing cGAS DNA sensing to produce 2′3′cGAMP, which binds to stimulator of IFN genes (STING) on the endoplasmic reticulum (ER) and triggers downstream signaling to produce type I interferons (IFNs) and proinflammatory cytokines. In cells lacking G3BP1, following HSV-1 infection or HSV-60 transfection, phosphorylation of STING and the downstream signaling by TBK1 and IRF3 and the ER stress effector eIF2α is attenuated (Figure 1A,B). To determine the role of G3BP1 in cGAMP-mediated STING activation, downstream of cGAS, HT1080 WT, G3BP1 KO and STING KO cells were transfected with 2′3′ cGAMP and the activation of the immune response by the phosphorylation of STING, TBK1 and IRF3 and ER stress by peIF2α was analyzed on immunoblots. Compared to WT cells, G3BP1 KO cells showed a significant reduction in pSTING (ratio of pSTING/tSTING was 24 in WT vs. 3.2 in G3BP1KO) and quantitative decrease in pTBK1 (WT vs. G3BP1 KO ratio of pTBK1/tTBK1 1.0 vs. 0.8), pIRF3 (WT vs. G3BP1 KO ratio of pIRF3/tIRF3 0.6 vs. 0.4) and peIF2α (WT vs. G3BP1 KO ratio of peIF2α/teIF2α 0.8 vs. 0.4) in 2′3′cGAMP transfected cells, while activation of the pathways was not observed in STING KO cells (Figure 1C). To further demonstrate that G3BP1 participated in STING activation independent and downstream of cGAS, we used a G3BP1 inhibitor, EGCG, and compared the phosphorylation of STING in WT cells transfected with HT-DNA and HSV-60, which are sensed by cGAS and activate cGAS/STING signaling, or with 2′3′cGAMP, which directly binds and activates STING independent of cGAS. Inhibiting G3BP1 reduced the phosphorylation of STING by 2′3′cGAMP, suggesting a role in regulating STING activity downstream of cGAS. As expected, EGCG inhibited pSTING in HT-DNA- and HSV-60-transfected cells (Figure 1D). The canonical STING signaling cascade, acting via TBK1-IRF3 and NF-κB, induces the transcription of type I IFN and proinflammatory cytokines. To determine if G3BP1 regulated this pathway when STING was directly activated by 2′3′cGAMP, we compared the transcriptional induction of *IFNβ*, *ISG56*/*P56*/*IFIT, CCL5*, and *IP-10* in WT, G3BP1 KO, and STING KO cells following transfection with 2′3′cGAMP and HSV-60, and infection with HSV-1. The transcriptional induction in response to infection with HSV-1 or introducing HSV-60 was reduced in cells lacking G3BP1 or STING (Figure 1E,F), consistent with the immunoblot analysis (Figure 1A,B). Interestingly, the type I IFN and cytokine induction was significantly reduced in cells lacking G3BP1, like STING KO cells transfected with 2′3′cGAMP (Figure 1G). The decrease in type I IFN production was reflected in the loss of the antiviral effect and increase in HSV-1 viral titers in cells lacking G3BP1 or STING compared to WT cells (Figure 1H). To further assess the role of G3BP1 in directly regulating STING activity, mediated by its oligomerization, we used a split Venus assay to visualize and quantify STING activation in live cells in response to dsDNA. WT and G3BP1 KO cells were transfected with STING plasmids fused to two non-fluorescent fragments of the Venus fluorescent protein, followed by transfection with 2′3′cGAMP or dsDNA pdA:dT. Compared to WT cells, we observed a decrease in the fluorescence emission by reconstituted Venus on the STING oligomerization induced by 2′3′cGAMP or pdA:dT in G3BP1 KO cells (Figure 1I). These findings suggest that G3BP1 has additional roles in regulating 2′3′cGAMP-induced STING activity beyond its role in facilitating enhanced cGAS activation [48,49].

### 3.2. G3BP1-STING Axis Coordinates ER Stress Response by DNA Virus and Viral DNA

**Figure 2:**
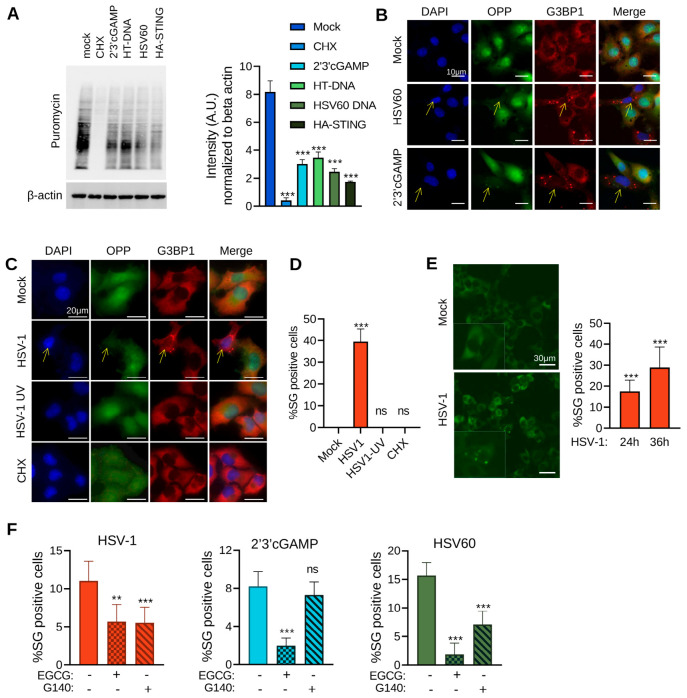
G3BP1-STING axis coordinates the ER stress response. (**A**) HT1080 cells were transfected with 2′3′-cGAMP (5 μg/mL), HSV60 (5 µg/mL), or HT-DNA (4 µg/mL) for 6 h or with the HA-STING plasmid (24 h), and cells were pulsed with puromycin (10 μg/mL) for 30 min and cell lysates were analyzed for puromycin-incorporated proteins on immunoblots using anti-puromycin antibody and normalized to β-actin. Mock-transfected cells or those treated with cycloheximide (CHX, 50 µg/mL) for 20 min followed by puromycin treatment were used as controls. Densitometric analysis was performed using ImageJ to quantify the incorporation of puromycin, and the values were normalized to control conditions and β-actin and plotted. *p* values in A were calculated by one-way ANOVA with Dunnett’s multiple-comparisons test compared to mock-transfected cells. HT1080 cells were seeded on glass coverslips and treated with (**B**) HSV-60 (5 μg/mL) or 2′3′cGAMP (5 μg/mL) for 6 h, or (**C**) infected with HSV−1 or UV-inactivated HSV−1 at MOI = 1.0 for 24 h, or treated in parallel with cycloheximide (CHX, (50 µg/mL)), and the levels of newly synthesized proteins in cells were visualized by Click-iT OPP Alexa Fluor 488 imaging, as described in the Methods, and with G3BP1 antibodies to stain SGs, followed by immunofluorescence analysis. Random images were captured, and further analysis and processing of images were performed using ImageJ software (v1.5) or Olympus Stream View software V2.3 (Olympus Corporation, Tokyo, Japan). Incorporation of OPP as an indicator of protein translation (green) was compared to cells forming SG puncta (red), and compared to mock-treated cells for 2′3′ cGAMP and HSV60 and uninfected and UV-inactivated HSV−1 for HSV−1-infected cells. The percentages of SG-containing cells (red) and those showing reduced OPP incorporation (green) are indicated by arrows. (**D**) The %SG-positive cells in HSV−1-infected cells, UV-inactivated HSV−1, CHX and uninfected cells were calculated in at least five random fields from a minimum of 100 cells and plotted as mean ± SD. *p* values in (**D**) were calculated by one-way ANOVA with Dunnett’s multiple-comparisons test compared to uninfected cells. HT1080 cells stably expressing GFP-G3BP1 were (**E**) infected with HSV−1 (MOI = 1) and at the indicated times, the percentage of GFP-positive cells forming SG puncta were calculated; and (**F**) infected with HSV−1 (MOI = 1.0) for 24 h, or transfected with 2′3′cGAMP (5 μg/mL) for 6 h or HSV-60 (5 μg/mL) for 6 h, in cells that were not treated or treated with either EGCG (20 μM) or G140 (20 μM), and the percentage of GFP-positive cells forming SG puncta were calculated. For (**E**), *p* values were calculated by Welch’s *t*-tests, comparing each HSV−1 time point with uninfected/mock values, with Holm correction across the two comparisons. For (**F**), *p* values were calculated by one-way ANOVA with Dunnett’s multiple-comparisons test, compared to cells not treated with inhibitors. SG analysis was performed in at least five random fields from a minimum of 100 cells per treatment from three independent experiments and plotted as mean ± SD. ** *p* < 0.01; *** *p* < 0.001, ns not significant. Scale bars, 10 µm, 20 µm or 30 µm as indicated.

STING activates a non-canonical ER stress pathway in response to DNA damage agents and immunostimulatory DNA by phosphorylating eIF2α and the formation of stress granules [34,35]. We observed an increase in peIF2α by HSV−1, HSV−60 and cGAMP in WT cells compared to G3BP1 KO cells (Figure 1A–C). To determine the role of the G3BP1-STING axis in modulating ER stress during DNA virus infection and in response to viral DNA, we first compared the impact on protein synthesis using a puromycin incorporation assay in HT1080 cells transfected with HT-DNA, HSV-60, 2′3′cGAMP or cells overexpressing the HA-STING plasmid, known to activate STING, compared to cycloheximide (CHX) as a positive control for translation inhibition. Treated cells were pulsed with puromycin that is incorporated into newly translated polypeptides and terminates translation, and cell lysates were analyzed on immunoblots using anti-puromycin antibodies. The levels of puromycin incorporated into proteins decreased 2.5–5-fold when STING was activated, and more importantly, directly by 2′3′cGAMP or HA-STING (Figure 2A). One of the consequences of translation arrest by eIF2α phosphorylation is the induction of SGs. We correlated the impact of the decrease in puromycin incorporation, reflective of reduced translation, on SG formation by measuring the incorporation of O-propargyl-puromycin (OP-Puro) using Click chemistry and staining SGs with anti-G3BP1 antibodies and visualizing them by immunofluorescence microscopy. In cells transfected with HSV-60 or 2′3′cGAMP, cells showing low OPP incorporation showed G3BP1 SG puncta (Figure 2B). HSV−1 infection also resulted in a decrease in OPP incorporation compared to no infection, and the decrease correlated with the formation of SG puncta stained with G3BP1. Viral replication in infected cells was required for this effect, as UV-inactivated HSV−1 did not inhibit translation or form SG puncta (Figure 2C,D). We then quantitated the SG formation in HT1080 cells stably expressing GFP-G3BP1 infected with HSV−1 (MOI = 1) at the indicated times from random fields. We observed an increase in % cells showing SGs at 24 and 36 h (Figure 2E), while UV-inactivated HSV−1 did not induce any SGs, as shown in Figure 2D. We analyzed SG formation induced by 2′3′cGAMP, HSV-60 or HSV−1 in cells when cGAS activity is inhibited by inhibitor G140, or G3BP1 is inhibited by treating with EGCG. 2′3′cGAMP activates STING downstream of cGAS, and as expected, inhibiting cGAS with G140 had a minimal effect on SG formation, while G3BP1 inhibition reduced SG activity. However, SGs induced by both HSV-60 viral DNA or by HSV−1 were inhibited by both inhibitors, with EGCG having a more drastic effect with HSV-60 than with HSV−1 (Figure 2F). These results show an important role of the G3BP1-STING axis in coordinating ER stress by regulating protein translation and stress granule formation, and demonstrates a role of G3BP1 when STING is activated directly by its ligand vs activating the cGAS-STING signaling cascade.

### 3.3. STING Activates PERK to Integrate Cellular Stress and Immune Signaling

**Figure 3:**
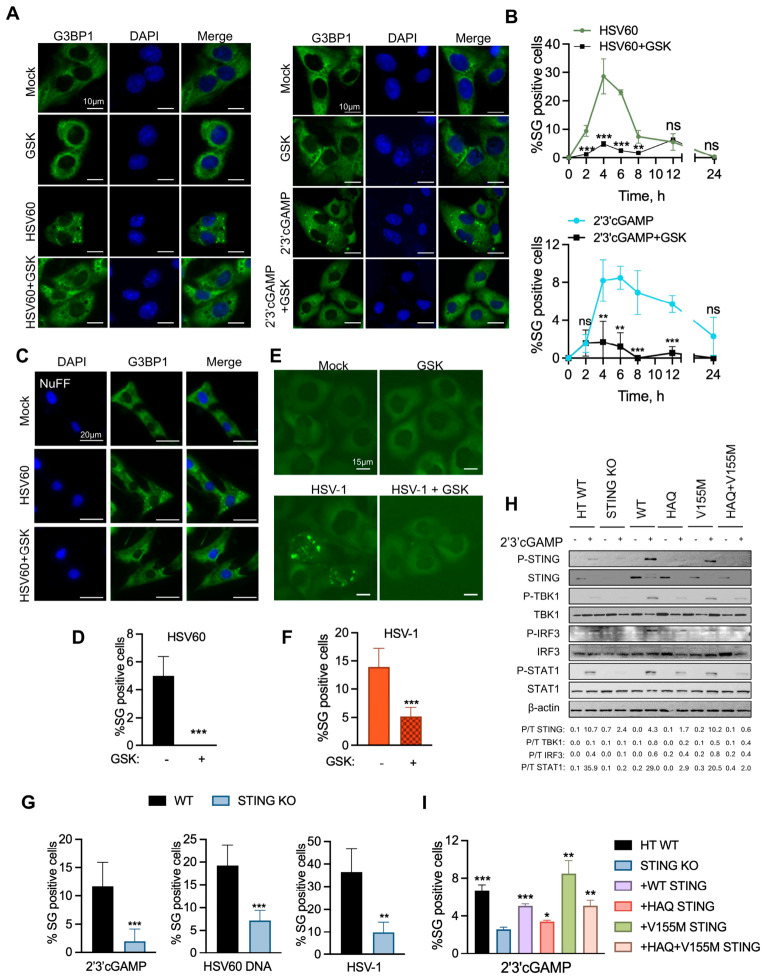
STING integrates the ER stress response through PERK. (**A**) HT1080 cells were treated with HSV−60 (5 μg/mL) or 2′3′cGAMP (5 μg/mL) for 6 h, without or with the PERK inhibitor (GSK2656157, GSK, 5 µM). (**B**) Kinetics of SG formation in HT1080 cells stably expressing GFP-G3BP1 treated with indicated DNA ligands without or with the PERK inhibitor (GSK, 5 µM), and (**C**,**D**) primary NuFF cells treated with HSV−60 (5 μg/mL), without or with the PERK inhibitor (GSK, 5 µM) for 6 h; SGs were immunostained with anti-G3BP1 (green) and DAPI (nuclei, blue), and SG-positive cells were analyzed by confocal microscopy (**A**,**C**) and quantitated (**D**), or the % of cells showing GFP-G3BP1-positive foci in the time course was determined (**B**) at the indicated time points from at least 100 cells from three replicates. Results are mean ± SD. *p* values in (**B**) were calculated by two-way ANOVA with Holm–Sidak-corrected planned comparisons between GSK-treated and untreated cells at each time point. (**E**) HT1080 cells stably expressing GFP-G3BP1 were infected with HSV−1 (MOI = 1) for 24 h in cells without or with the PERK inhibitor (GSK, 5 µM) and SG-positive cells were analyzed, and (**F**) the percentage of GFP-positive cells forming SG puncta were calculated. *p* values in D and F were calculated by Welch’s *t*-tests comparing cells treated without or with GSK. (**G**) HT1080 WT and STING KO were transfected with 2′3′cGAMP (5 μg/mL) or HSV−60 (5 μg/mL) or infected with HSV−1 (MOI = 1.0), and SGs were immunostained with anti-G3BP1, and SG-positive cells were quantitated. The percentage of cells showing G3BP1-positive SG puncta was determined from at least 100 cells from three replicates. Results are mean ± SD. *p* values in G were calculated by Welch’s *t*-tests compared to WT cells. HT1080 WT, STING KO and STING KO cells stably expressing WT STING, HAQ, V155M or V155M HAQ combined were mock-transfected or transfected with 2′3′cGAMP (5 μg/mL), (**H**) cell lysates were analyzed for the phosphorylation of STING, TBK1, IRF3 and STAT1 and compared to total protein levels, and loading was normalized to β-actin levels. The ratios of the indicated phospho-proteins/total proteins for the antibodies probed were quantified by ImageJ. (**I**) SGs were immunostained with anti-G3BP1 and SG-positive cells were quantitated. The percentage of cells showing G3BP1-positive SG puncta was determined from at least 100 cells from three replicates. Results are mean ± SD. *p* values in I were calculated by one-way ANOVA with Dunnett’s multiple-comparisons test compared to STING KO cells. SG analysis was performed in at least five random fields from a minimum of 100 cells per treatment from three independent experiments and plotted as mean ± SD. * *p* < 0.05; ** *p* < 0.01; *** *p* < 0.001, ns not significant. Scale bars, 10 µm, 15 µm or 20 µm, as indicated.

Among the stress kinases that phosphorylate eIF2α, causing a subsequent reduction in protein synthesis, the ER stress kinase, PERK, is a direct target of STING signaling [34,35,51,52]. To determine if the STING-induced peIF2α protein synthesis inhibition and SG induction in response to 2′3′cGAMP and HSV-60 is mediated by PERK, HT1080 GFP-G3BP1 cells were pretreated with PERK inhibitor GSK2656157 (GSK) or mock-treated followed by 2′3′cGAMP or HSV-60, and SG formation in real time was monitored. SG formation was observed in ligand-treated cells, while inhibiting PERK activity resulted in the loss of SG accumulation (Figure 3A). The dynamic assembly/disassembly of SGs over time with ligand treatment was inhibited in cells treated with the GSK inhibitor (Figure 3B). In primary NuFF cells (human newborn foreskin fibroblasts) transfected with HSV-60, we observed a similar induction of SGs which was inhibited by GSK pretreatment, extending the relevance of our observations to primary cells (Figure 3C,D). SG formation in response to HSV−1 infection was inhibited in GFP-G3BP1-expressing cells with the PERK inhibitor (Figure 3E,F). We then tested the role of STING as the mediator of the ER stress response by comparing SG formation in WT and STING KO cells transfected with 2′3′cGAMP or HSV-60 or infected with HSV−1. Reduction in SG formation, like with GSK-treated cells, was observed in STING KO cells, indicating an important role of STING in coordinating the ER stress response (Figure 3G). To further test the features of STING that may be required for inducing the immune response and stress response, we reconstituted STING KO cells with (a) WT STING, (b) HAQ, triple mutant R71H-G230A-R293Q, defective in ligand binding and functioning as null, (c) V155M, which constitutively activates STING and (d) STING V155M HAQ combined, which has defective signaling and remains in the ER [53], and transfected the cells with 2′3′cGAMP to activate STING and analyzed the phosphorylation of downstream effectors by immunoblot analysis (Figure 3H). Expression of WT STING restores STING activity when treated with 2′3′cGAMP, and activation of the TBK1-IRF3 signaling cascade as well as SG formation. The HAQ mutant showed significantly reduced phosphorylation of the effectors of the STING signaling axis and SGs, while the constitutively active V155M mutant showed an increase in signaling and significantly more SG formation. In the HAQ/V155M mutant, immune signaling was compromised and the expression of HAQ dampened the effect of constitutive STING in SG formation, but the levels were greater than HAQ alone (Figure 3I). These findings demonstrate an important role of STING in regulating the ER stress response through PERK, in addition to the canonical immune signaling in response to the DNA virus and viral DNA.

### 3.4. PERK Activity Is Required for IFNβ and Cytokine Induction and SG Formation in Response to Viral DNA and cGAMP

**Figure 4:**
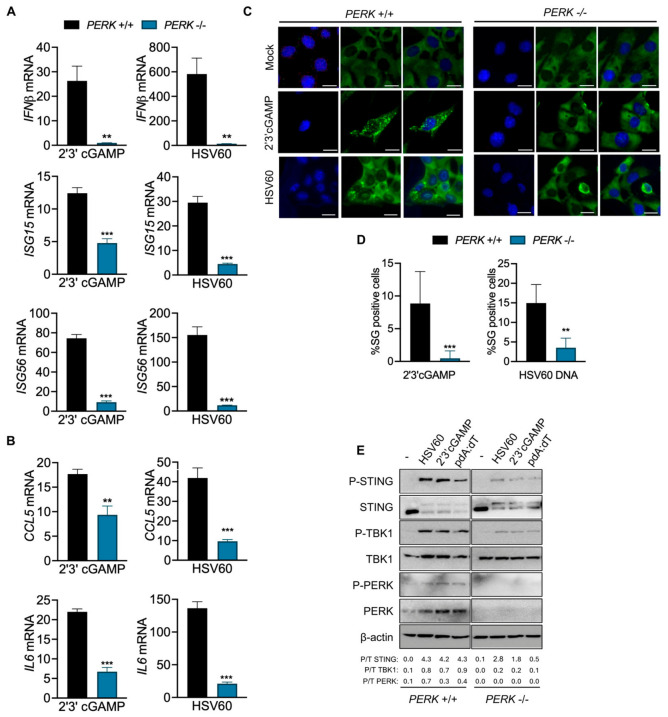
Role of PERK in cGAS-STING signaling in response to viral DNA and cGAMP. *PERK*^+/+^ and *PERK*^−/−^ MEFs plated in three independent wells were transfected with 2′3′cGAMP (5 μg/mL) and HSV-60 (5 μg/mL), and the mRNA levels of (**A**) *IFNβ*, *ISG5*6 and *ISG15* and (**B**) *IL6* and *CCL5* were measured by qRT-PCR and normalized to GAPDH mRNA levels and plotted as fold induction. Data represents the mean ± SE from three independent experiments. (**C**,**D**) *PERK*^+/+^ and *PERK*^−/−^ MEFs were transfected with 2′3′cGAMP (5 μg/mL) and HSV-60 (5 μg/mL), cells were fixed after 6 h and SGs immunostained with anti-G3BP1 (green) and DAPI (nuclei, blue), and SG-positive cells were analyzed by confocal microscopy and quantitated. The percentage of cells showing G3BP1-positive SG puncta was determined from at least 100 cells from three replicates. Results are mean ± SD. *p* values in (**A**,**B**,**D**) were calculated by Welch’s *t*-tests compared to *PERK*^+/+^ cells under the matching stimulus. (**E**) *PERK*^+/+^ and *PERK*^−/−^ MEFs were transfected with 2′3′cGAMP (5 μg/mL), HSV-60 (5 μg/mL), or pdA:dT (5 μg/mL) for 6 h, cell lysates were analyzed for the phosphorylation of STING, TBK1 and PERK and compared to total protein levels, and loading was normalized to β-actin levels. The ratios of the indicated phospho-proteins/total proteins for the antibodies probed were quantified by ImageJ. ** *p* < 0.01; *** *p* < 0.001.

To determine the impact of PERK in mediating the gene induction of IFN and proinflammatory cytokines when STING is activated, *PERK*+/+ and *PERK*−/− MEFs were transfected with 2′3′cGAMP or HSV-60 and mRNA levels were compared. Transcriptional induction of *IFNβ* in response to both 2′3′cGAMP and HSV-60 was PERK-dependent, as minimal induction was observed in *PERK*−/− MEFs. Concomitant increases in mRNA levels of the interferon-stimulated genes *ISG56*/*P56*/*IFIT1* and *ISG15* were observed in *PERK*+/+ MEFs. NF-κB signaling is another pathway downstream of STING signaling which mediates cytokine induction, in addition to the canonical activation of the TBK1-IRF3 axis [17]. As with *IFNβ* and ISG induction, mRNA levels of the cytokines *CCL5* and *IL-6* were also significantly higher in *PERK*+/+ cells compared to *PERK*−/− cells (Figure 4A,B). Inhibiting PERK activity as we showed above, resulted in a decrease in SG formation when STING is activated directly by 2′3′cGAMP or by viral DNA HSV-60. We performed immunofluorescence assays to detect G3BP1 in SGs formed in MEF cells mock-treated or treated with 2′3′cGAMP or HSV-60. Consistent with our results using the PERK inhibitor, GSK, we observed fewer SGs formed in MEFs lacking PERK treated with either 2′3′cGAMP or HSV-60 (Figure 4C,D). Interestingly, cell lysates from *PERK*−/− MEFs treated with various dsDNA ligands and 2′3′cGAMP showed reduced phosphorylation of STING that correlated with the decrease in pTBK1 compared to *PERK*+/+ MEFs (Figure 4E). The results suggest that PERK activated by STING is required for the induction of type I IFN and cytokines, as well as SG formation. The reduced pSTING levels in *PERK*−/− MEFs suggests that PERK may also regulate STING in a reciprocal mechanism that will need further investigation.

### 3.5. G3BP1 Potentiates cGAMP-Induced STING Trafficking from the ER to Golgi

**Figure 5:**
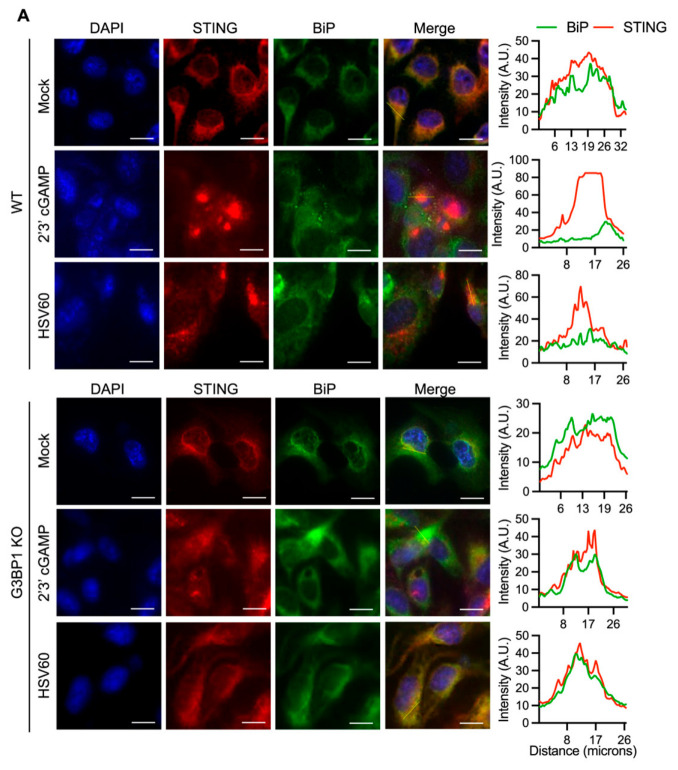

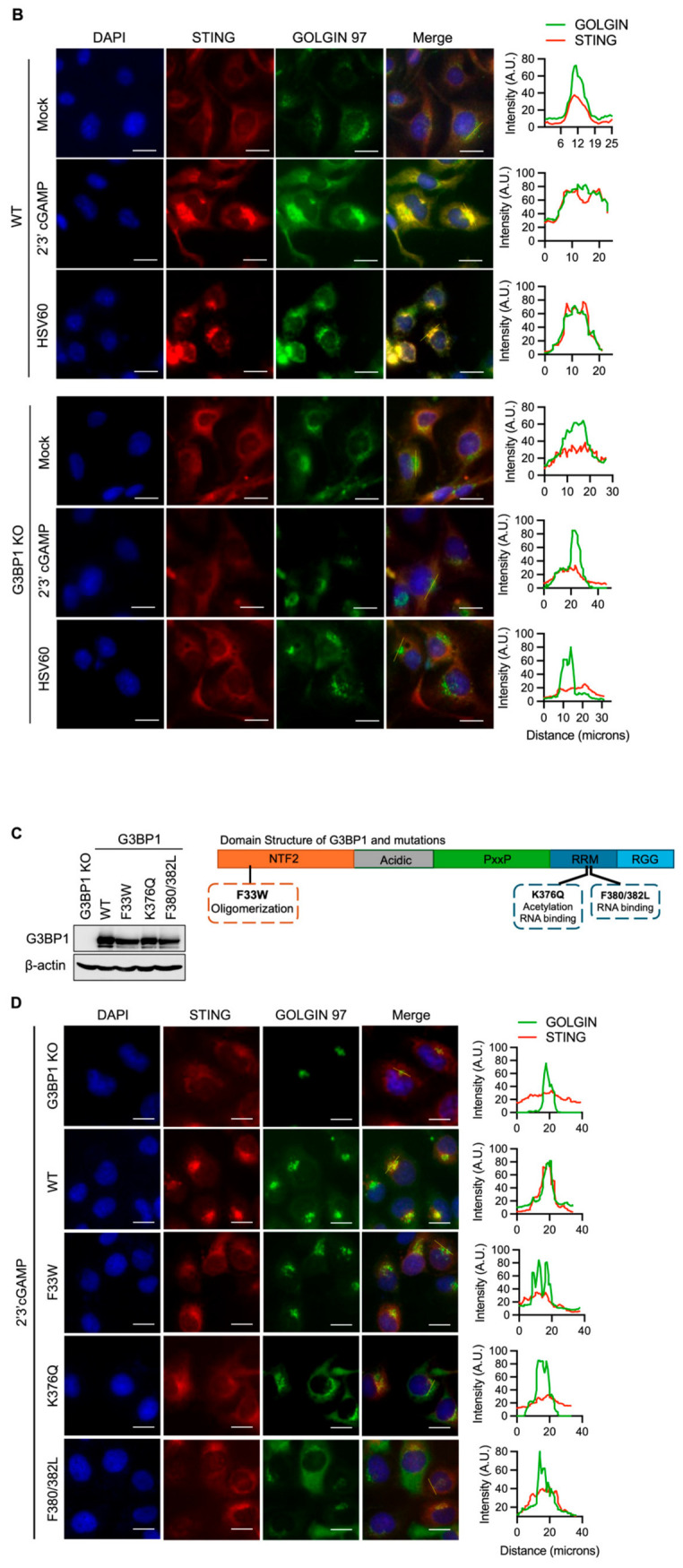

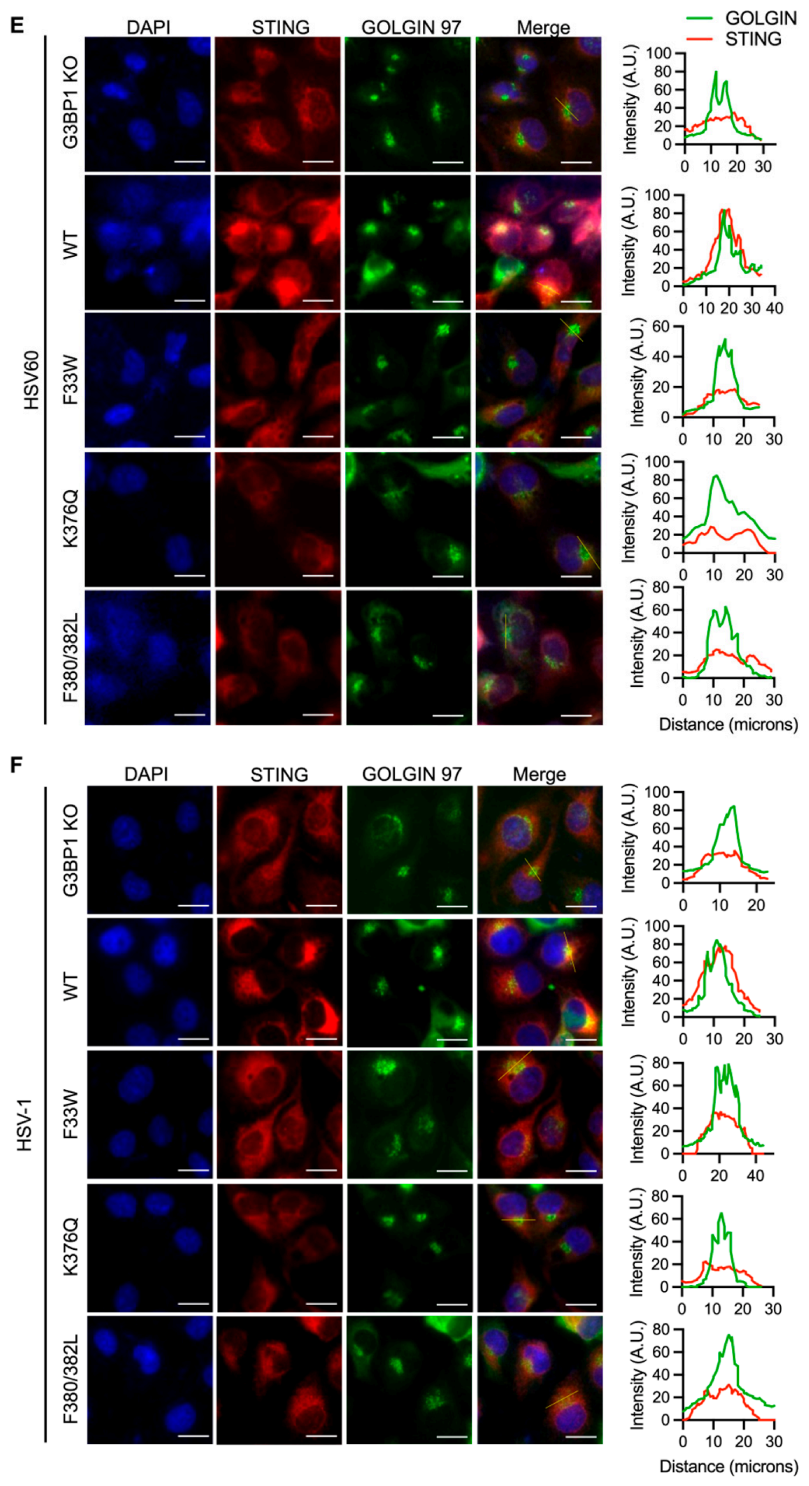
G3BP1 regulates STING trafficking from the ER to Golgi. HT1080 WT and G3BP1 KO cells were plated on coverslips in triplicate and after 24 h, cells were mock-transfected, or transfected with 2′3′cGAMP (5 μg/mL) or HSV-60 (5 μg/mL) for 6 h and fixed as described in the Methods. Cells were stained with (**A**) anti-STING (red) and anti-BiP (green), an ER marker, and DAPI (nuclei, blue), (**B**) anti-STING (red) and anti-golgin97 (green), a Golgi marker, and DAPI (nuclei, blue), and analyzed by confocal microscopy. (**C**) Schematic of domains of full-length WT and G3BP1 mutants and expression levels in G3BP1 KO stable cells of WT and G3BP1 mutants on immunoblots, normalized to β-actin levels. G3BP1 KO cells and stable cells expressing WT, F33W, K376Q or F380/382L were plated on coverslips and after 24 h, cells were transfected with (**D**) 2′3′cGAMP (5 μg/mL) or (**E**) HSV-60 (5 μg/mL), or (**F**) infected with HSV−1 (MOI = 1.0). Cells were fixed and stained with anti-STING (red) and anti-golgin97 (green), a Golgi marker, and DAPI (nuclei, blue), and analyzed by confocal microscopy. Line scan analysis was performed along the indicated lines in the merged image to assess the colocalization (separate colors shown on graphics) of markers using ImageJ. A minimum of five random fields were analyzed and representative images are shown. Scale bars, 20 μm.

Activation of STING directly by 2′3′cGAMP, by viral dsDNA or the DNA virus, activates the canonical IFN signaling pathway and production of cytokines, and regulates protein translation and SG formation, and our results demonstrate that G3BP1 is required to not only serve as a co-factor and activator of cGAS at the DNA sensing level, but also directly regulate STING activity downstream of cGAS. We previously demonstrated that overexpression of STING in G3BP1 KO cells showed reduced pSTING and IFN induction that correlated with diffuse staining of STING in the ER, indicating that G3BP1 may regulate the STING ER to Golgi translocation [35]. We first transfected WT and G3BP1 KO cells with 2′3′cGAMP or HSV-60 and used immunofluorescence microscopy to determine the distribution of STING with the ER marker, BiP, or with the Golgi marker, Golgin97. In untreated cells, STING is localized in the endoplasmic reticulum (ER), evident from colocalization with BiP. Upon activation, in WT cells, STING translocates to the Golgi and aggregates as distinct puncta in the perinuclear region that colocalizes with Golgin97. However, in G3BP1 KO cells, treatment with 2′3′cGAMP or HSV-60 did not alter STING staining in the ER and did not form dense puncta as in WT cells (Figure 5A,B). G3BP1 is a nucleator of SGs in response to diverse stress stimuli, and we previously demonstrated that TIA1, another component of SGs, colocalizes with G3BP1 in SGs formed by cytosolic dsDNA. WT and TIA1 KO cells were mock-transfected or transfected with 2′3′cGAMP, and SG formation was quantitated (Supplementary Figure S1A). We observed no significant difference in SG formation in cells lacking TIA1, which was further confirmed by immunofluorescence staining of SGs by G3BP1 (Supplementary Figure S1B). To test if TIA1, like G3BP1, may have broader roles in regulating STING trafficking, WT and TIA1 KO cells were treated with 2′3′cGAMP, and we used immunofluorescence microscopy to determine the translocation of STING to the Golgi using golgin 97. In untreated cells, STING is diffuse and localized in the ER, and both WT and TIA1 KO cells, upon activation with 2′3′cGAMP, show STING aggregates as distinct dense puncta colocalizing with golgin 97 in the perinuclear region, characteristic of oligomerization and translocation (Supplementary Figure S1C). Lack of TIA1 did not abrogate SG formation; however, G3BP1 KO cells do not form any SGs, demonstrating its essential role in SGs. Furthermore, cells lacking G3BP1 treated with cGAMP showed reduced STING activity (Figure 1), suggesting that the role of G3BP1 in inducing SGs may be distinct from its role in regulating STING trafficking, and based on these results, we are unable to segregate the role of SGs in regulating STING. These results further support our data that G3BP1 has a distinct role, in addition to its canonical role in SG nucleation.

To determine if the activity of PERK is required for STING trafficking downstream of cGAS, PERK+/+ and PERK−/− MEFs were transfected with 2′3′cGAMP and the distribution of STING with the ER marker, BiP, was compared by immunofluorescence microscopy. In untreated cells, STING is localized in the endoplasmic reticulum (ER) based on colocalization with BiP, and on activation, STING forms aggregates in the perinuclear region, similar to our observation in HT1080 cells in Figure 5. We did not observe any difference between PERK+/+ and PERK−/− MEFs, indicating that PERK is not required for STING translocation on activation by 2′3′cGAMP (Supplementary Figure S2).

To mechanistically determine the role of G3BP1 in modulating STING, we generated G3BP1 mutants impacting the predicted roles of G3BP1: (a) F33W oligomerization-defective, (b) K376Q acetylation- and RNA-binding-defective and (c) F380/382L RNA binding- and SG-defective, and generated stable cell lines in G3BP1 KO cells (Figure 5C). To determine the role of the G3BP1 mutants in SG formation, we transfected the stable cell lines with synthetic dsRNA, polyI:C, known to induce SGs, or with HSV-60 or 2′3′cGAMP, and quantitated SG formation. We used dsRNA in these experiments as we can exclude the role of cGAS-mediated enhancement and characterize the well-established role of G3BP1 as a nucleator of SGs. As expected, oligomerization (F33W) and acetylation (K376Q)-defective mutants significantly reduce SG formation, while the RNA-binding defective double mutant (F380/382L) is severely impaired in SG formation (Supplementary Figure S3A–C). We transfected the G3BP1 KO and reconstituted cells with 2′3′cGAMP or HSV-60 and checked the localization of STING with the Golgi marker, Golgin, by immunofluorescence microscopy. Expression of WT G3BP1 in KO cells facilitated the aggregation of STING and transit to the Golgi in response to both 2′3′cGAMP and HSV-60. G3BP1 mutants were defective to variable extents in restoring STING aggregation or translocation with both ligands (Figure 5D,E). As the effect of 2′3′cGAMP is downstream of cGAS, the effects of G3BP1 we observed were predicted to be directly targeting STING activity. The inability of G3BP1 mutants, defective in oligomerization and protein interaction, RNA-binding and signaling, to promote STING signaling suggests that multiple parts of G3BP1 participate in coordinating STING function. In other studies, G3BP1 deletion constructs missing various domains were uniformly defective in promoting cGAS DNA binding capacity [48]. We then tested the role of G3BP1 mutants in STING trafficking during HSV−1 infection (Figure 5F). As with HSV-60, cells lacking G3BP1 or expressing G3BP1 mutants lacked STING puncta and showed similar defects and retention of STING in the ER (Figure 5F). Our results suggest an important role of G3BP1 in regulating STING activity by modulating its trafficking from the ER to Golgi in response to activation by ligands or DNA virus infection.

### 3.6. G3BP1 Mutants Defective in STING Trafficking Reduce Cytokine Induction and Antiviral Effect

**Figure 6:**
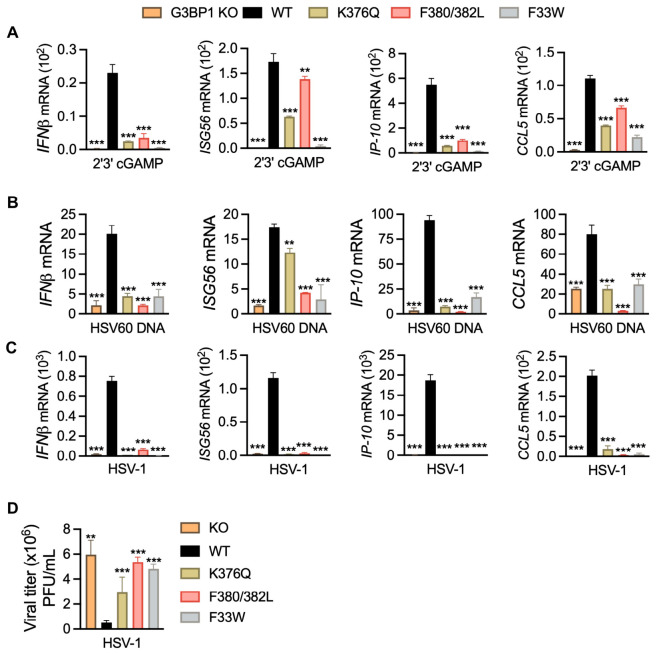
Effect of G3BP1 mutants on STING-induced IFN and cytokine production and the HSV−1 antiviral effect. G3BP1 KO cells and stable cells expressing WT, F33W, K376Q or F380/382L were plated in three independent wells and were transfected with (**A**) 2′3′cGAMP (5 μg/mL) or (**B**) HSV-60 (5 μg/mL) for 6 h or (**C**) infected with HSV−1 (MOI = 1.0), and the mRNA levels of IFNβ, ISG56, IP-10 and CCL5 were measured by qRT-PCR and normalized to GAPDH mRNA levels and plotted as fold induction. Data represents the mean ± SE from three independent experiments. *p* values were calculated by one-way ANOVA with Dunnett’s multiple-comparisons test compared to G3BP1 KO cells expressing WT protein. (**D**) G3BP1 KO cells and stable cells expressing WT, F33W, K376Q or F380/382L were plated in three independent wells and infected with HSV−1 (MOI = 1.0), and after 24 h, the virus titer in supernatants was determined by plaque assay. Data shown are from experiments performed in triplicate and at least repeated twice; the graph shows mean ± SD, *n* = 3. *p* values were calculated by one-way ANOVA with Dunnett’s multiple-comparisons test compared to G3BP1 KO cells expressing WT protein. ** *p* < 0.01; *** *p* < 0.001.

STING, on binding cGAMP, undergoes oligomerization and translocates to the Golgi, which is required for its activation and downstream signaling for inducing type I IFN and cytokines. Based on our data that G3BP1 modulates STING trafficking, we investigated the effect of G3BP1 mutants in the transcriptional induction of type I IFN, ISG and cytokines in response to 2′3′cGAMP, HSV-60 and HSV−1 infection. Induction of *IFNβ* mRNA was significantly reduced in cells expressing G3BP1 mutants compared to the robust induction in WT G3BP1-expressing cells; the impact was very apparent in HSV−1-infected cells (Figure 6A–C). Broadly, *ISG56*/*P56*/*IFIT1* induction is lower than WT, and the difference with some of the mutants may be reflected by an established IRF3-independent induction that is induced by the ISGF3 complex that includes IRF9, STAT1 and STAT2. Recent studies showed that STAT2 may regulate STING trafficking, and further studies may provide more insights into the mechanistic details [54]. The induction of NF-κB target genes and cytokines by STING was restored by the expression of WT G3BP1, while the G3BP1 mutants showed an attenuated response (Figure 6A–C). Our results suggest that G3BP1 KO or mutant cells which have reduced IFNβ production would show a loss of the antiviral effect against DNA virus infection. To test this, G3BP1 KO and cells reconstituted with G3BP1 mutants were infected with HSV−1 at an MOI of 1.0, and the viral titers in culture supernatants were determined. In line with our hypothesis, the viral titer was low in WT G3BP1-expressing cells, while G3BP1 mutants that produced less IFNβ had a higher viral load (Figure 6D). The distinct lack of IFN, ISG and cytokine induction in the absence of functional G3BP1 during HSV−1 infection supports the role played by G3BP1 at multiple levels: enhancing cGAS activity and STING translocation.

## 4. Discussion

**Figure 7:**
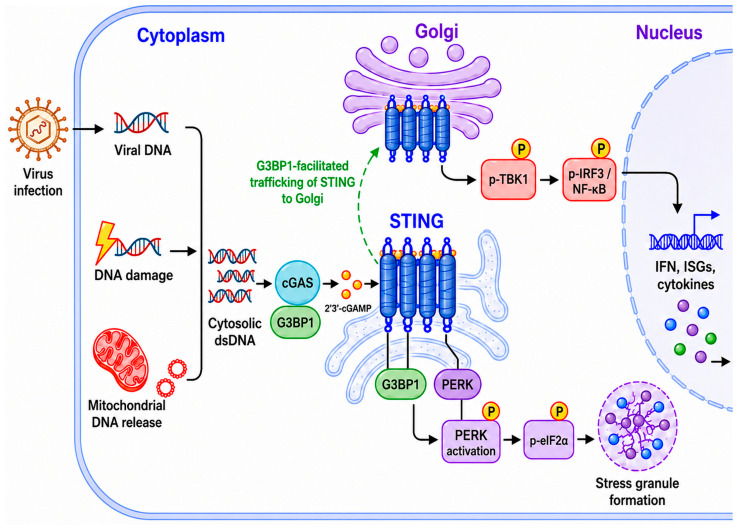
A proposed model for the multiple roles of G3BP1 in regulating cGAS-STING and the integrated stress response pathway. cGAS senses aberrant cytosolic self or pathogenic DNA and synthesizes 2′3′cGAMP, which binds and activates STING. Activated STING oligomerizes and translocates to the Golgi, where phosphorylation of TBK1 and IRF3 activates type I IFN and cytokine production. G3BP1 regulates multiple steps of the cGAS-STING pathway by enhancing cGAS DNA binding, STING activity and regulating the translocation of STING to the Golgi to trigger IFN and cytokine production. STING activates the ER stress response by PERK and phosphorylation of eIF2α, resulting in SG formation. Recent studies show STING-G3BP1 interaction regulates SG precondensation and maturation, and STING-PERK interaction regulates the integrated stress response. See the Discussion for more details.

The cGAS/STING pathway is activated by aberrant cytosolic dsDNA and cGAS activity produces 2′3′cGAMP, which binds to and activates the ER adaptor, STING (Figure 7). STING functions canonically as a mediator in innate immunity and inflammation by triggering type I IFN production via TBK1-IRF3, and proinflammatory cytokines via NF-κB, and non-canonically functioning as a proton channel promoting autophagy and activating PERK-eIF2α pathways. STING activity is regulated in cells on binding 2′3′cGAMP, inducing a conformational change and oligomerization, promoting translocation from the ER to Golgi. Phosphorylation of STING, TBK1 and IRF3 activates the downstream signaling events, and the subsequent Golgi to lysosome transport of STING facilitates degradation, terminating its activity. STING activity can therefore be regulated at multiple levels, including upstream DNA sensing leading to cGAMP production, post-translational modifications and protein–protein interaction retaining STING at the ER or enhancing its exit, or by components of pathways that regulate vesicular transport and degradation [24]. Our study identifies a non-canonical role of G3BP1, a key marker of stress granules, by modulating intracellular STING trafficking from the ER to Golgi, in addition to its previously identified role in promoting the activation of cGAS to produce cGAMP and STING activation. In cells lacking G3BP1, both the activation of STING signaling by 2′3′cGAMP, which is downstream of cGAS, IFN and cytokine induction, and non-canonical activation of the ER stress response are impaired. Mechanistically, we show that G3BP1 facilitates STING translocation to the Golgi, as G3BP1 mutants show compromised STING aggregation and Golgi transit. In support of our findings, recent studies showed that the C-terminal domain of STING, which extends into the cytoplasm, interacts with G3BP1 to provide a scaffold for SG precondensation and maturation [36], while the STING at the ER interacts with PERK [34] to activate the ER stress response, and we demonstrate its role in SG formation. A limitation of our study is that we did not identify the dynamics of interaction of G3BP1-STING-PERK to ascribe the spatiotemporal steps in the pathway. Additionally, we were unable to segregate the signaling vs SG function of G3BP1 using genetic deletion of SG components like TIA1 or G3BP1 mutants due to overlapping functions. It should also be noted that the decrease in SG formation in STING KO cells (Figure 3G) and reduced pSTING in *PERK*^−/−^ MEFs (Figure 4E) support two-way reciprocal regulation and will be the key direction in future studies. Regardless, our studies reveal multiple roles for G3BP1 in innate immune signaling and the stress response by enhancing cGAS-STING activity and regulating STING trafficking, linking DNA sensing, signaling and cellular stress pathways with extended relevance to diseases associated with DNA damage and dysregulated stress responses (Figure 7).

Multiple dsDNA sensors are expressed in cells and reported to produce type I IFN; however, in genetic ablation experiments in vivo, only cGAS is essential to produce IFN through STING activation [3]. Recent studies show that cGAS activity is regulated by phase-separated condensates, and G3BP1, a SG-nucleating protein, promotes DNA binding and activation of cGAS [48,49,55]. G3BP1, in its canonical role, functions as a molecular switch that triggers liquid–liquid phase separation of RNA and RNA-binding proteins into dynamic stress granules that assemble and disassemble in response to stress stimuli, including virus infection and ER stress. Our data supports the requirement of G3BP1 to enhance cGAS activity and STING activation in cells treated with HSV-60, derived from HSV−1 genomic DNA, as well as HSV−1 infection. The phosphorylation of STING and the signaling cascade leading to IFNβ and cytokine production is significantly reduced in G3BP1-deficient cells. Unexpectedly, G3BP1-deficient cells show reduced STING signaling in response to direct activation of STING by 2′3′cGAMP, and because cGAMP bypasses cGAS, our observation places part of the G3BP1 defect at or downstream of STING, separate from the known role as a cGAS co-factor. Inhibiting G3BP1, but not cGAS, resulted in a decrease of SG formation by 2′3′cGAMP, while both G3BP1 and cGAS activity was required for SGs with HSV-60 and HSV−1 infection. These combined observations point to the roles of G3BP1 in regulating cGAMP-induced STING in IFN and cytokine signaling, as well as the ER stress response. In G3BP1KO cells treated with 2′3′cGAMP, the fact that there is a visible reduction in STING aggregation and persisting overlap with BiP in our data suggests G3BP1 facilitates the ER to Golgi STING translocation. In published studies, truncations across G3BP1 functional domains compromised cGAS DNA binding [48], so we generated amino acid substitutions in the full-length protein to reconstitute G3BP1 KO cells to define the STING trafficking features. As with cGAS activation, mutations in the functional domains of G3BP1 compromised the STING trafficking to the Golgi by 2′3′cGAMP, HSV-60 or HSV−1 infection, whereas WT G3BP1-expressing cells showed STING puncta in the Golgi that correlated with the activation of the canonical STING-TBK1-IRF3 axis. In line with this signaling defect, G3BP1 mutant cell lines showed a loss of the antiviral effect and HSV−1 replicated to higher titers. Translocation of STING from the ER to Golgi is essential for its downstream signaling functions, as inhibitors like Brefeldin A block the phosphorylation of TBK1 and IRF3 and innate signaling [9,19,20]. STING activity, stability, and trafficking are tightly regulated by post-translational modifications. For instance, S366 phosphorylation is required for IRF3 recruitment, and K63 ubiquitination (Ub) promotes trafficking to the Golgi and TBK1 interaction, while K48 ubiquitination is involved in a degradation of curb signaling, palmitoylation at Cys88 and Cys91 is required for membrane clustering and oligomerization, and SUMOylation blocks K48 Ub, keeping STING active [18,19,20,21,22,23,56,57]. Additionally, several proteins independent of vesicle biogenesis regulate STING vesicular trafficking by retaining at the ER or promoting Golgi transport, such as STIM1 (stromal interaction molecule 1), iRhom2 (inactive rhomboid protein 2), STEEP (STING ER exit protein) and NPC1 (Niemann Pick type C1) [58,59,60,61,62]. Recent studies identified STAT2 as a negative regulator of STING intracellular trafficking, wherein the STING E316 residue and the phosphorylation of STAT2 at T404 are both required to form the complex with STAT2 that blocks STING trafficking [54]. The many levels of fine-tuning STING trafficking emphasize the necessity of keeping the pathway in check in the context of human diseases and pathologies with aberrant DNA sensing and signaling.

STING is positioned at the ER and functions at the intersection of innate signaling and the ER stress response. Accordingly, we find that activation of STING, by dsDNA ligands, 2′3′cGAMP or by overexpression that is known to activate STING, results in a decrease in translation accompanied by peIF2α that serves as a trigger for SG assembly. Cells showing reduced translation, based on puromycin incorporation, form SGs in response to 2′3′cGAMP, HSV-60 and HSV−1 infection, compared to UV-inactivated HSV−1, indicating the requirement of virus replication. We showed previously that PERK, and not PKR, phosphorylates eIF2α in MEFs in response to 2′3′cGAMP, HSV-60 and other dsDNA ligands [35]. Accordingly, inhibiting PERK activity using pharmacological inhibitors in HT1080 or NuFF cells, or genetic ablation using MEFs, significantly reduces SG formation. The induction of IFN, ISGs and cytokines was also impaired in *PERK*−/− MEFs compared to *PERK*+/+ MEFs. We made a surprising observation that in *PERK*−/− MEFs, the phosphorylation of STING and TBK1 was reduced, indicating that PERK may directly phosphorylate STING or mediate STING phosphorylation by another kinase, and subsequently TBK1 phosphorylation is in a reciprocal relationship for “robust” activation. While we have not explored the interaction between STING and PERK, studies in senescence and fibrosis models have demonstrated direct interaction between the two proteins that is very transient and precedes TBK1-IRF3 activation [34]. Our data shows that PERK activity does not impact STING translocation (Supplementary Figure S2). During the preparation of this manuscript, a published study demonstrated that STING functions as a scaffold to promote SG formation by interacting with G3BP1 and UBAP2L via its cytoplasmic C-terminal domain, and the canonical pathway involving TBK1-IRF3 was dispensable [36]. In our study, STING mutants that were constitutively active induced signaling and formed a greater number of SGs compared to inactive mutants or cells lacking STING expression. These studies support our findings that G3BP1 function extends beyond enhancing DNA detection by cGAS, and the G3BP1-STING axis has additional roles in integrating the ER stress response, and these roles are distinct.

G3BP1 primarily functions to regulate RNA metabolism and translation during cellular stress; it also regulates the innate immune response to dsDNA via cGAS sensing, promoting the STING-TBK1-IRF3 pathway, STING translocation (this study) and as a core protein for stress granules during viral infection. HSV−1 expresses multiple viral proteins, especially immediate early (IE) proteins, tegument proteins, and other functional proteins that target cellular antiviral signaling pathways to block G3BP1-defense to ensure efficient replication [63,64,65,66]. HSV−1 DNA can be sensed during various steps of the viral life cycle. HSV−1 DNA in the early steps can be released into the cytoplasm following the degradation of the HSV−1 capsid for detection by DNA sensors and IFN production [63], or by leaked or exposed viral genomes during uncoating or replication, or when nucleocapsids exit the nucleus. The presence of cytosolic dsDNA can also be produced by HSV−1 infection as a product of mitochondrial damage and the release of mtDNA into the cytoplasm [64]. In our study, we speculate the sources of cytosolic HSV−1 genomic DNA could be due to any of these factors, and recognition would still be dependent on G3BP1 as a cGAS co-factor. Several HSV−1 proteins like ICP0, ICP8 and VHS (UL41) target G3BP1 to prevent the accumulation of SGs [64,66]. The endonuclease activity of vhs can also produce RNA breakdown products which can induce SG formation in a mechanism like the cleavage products of RNase L, a host endoribonuclease, which remains to be investigated. Unlike studies in the literature, we have monitored SG formation in live cells following HSV−1 infection, and our data supports the notion that SG formation varies dynamically over the course of infection, and further investigation is needed to demonstrate the events that can be conclusively attributed to SG induction. In addition, HSV−1 expresses UL37, VP22, VP1-2, UL36, ICP27, US3, and UL38 to target steps involved in cGAS sensing or STING-TBK1-IRF3 signaling to disable the host antiviral signaling pathways, establishing the importance of the cGAS-STING pathways in protecting the host cells [67]. Many of these proteins likely have temporal- and cell-type-specific roles and contribute differentially in HSV pathogenesis in vivo. Our observations, comparing the innate response of HSV-60 DNA and HSV−1 infection, provided insight uniquely into the mechanisms that underlie sensing the viral DNA in the absence of viral protein antagonism. The STING pathway provides a focal point from where multiple antiviral responses emerge, and we identified a role for G3BP1 in coordinating not only the IFN response, but also the ER stress response that is triggered by cellular stress due in part to the enhanced viral protein translation and host translation shut-off. Mutant viruses lacking some of these key viral proteins will be useful to demonstrate their role in restricting virus replication and contribute to our understanding of the complicated interplay of the intersecting pathways.

In summary, our study reveals a non-canonical role of G3BP1 in regulating the dsDNA-sensing pathway by impacting cGAMP-induced STING translocation from the ER to Golgi, a step that is required for the established role of STING in IFN and cytokine signaling. Several proteins and modifications on STING add to the complexities of the transport of STING, which emphasizes the need for the intricate control underlying STING activation and degradation. Dysregulated STING trafficking drives a wide spectrum of human pathologies like autoimmune diseases, neurodegenerative disorders, cancer immunotherapy, fibrosis and aging. Further investigations into STING activities will provide new insights into developing new therapeutic approaches for associated pathologies.

## Figures and Tables

**Table 1:** G3BP1 site-directed mutagenesis primer sequences.

Primer	Orientation	Sequence
G3 K376Q	F	5′ CAACAAAACCAAAATTGGGTAACTGCCCACCACTGTTAATGCGCAAC 3′
G3 K376Q	R	5′ GTTGCGCATTAACAGTGGTGGGCAGTTACCCAATTTTGGTTTTGTTG 3′
G3 F380/382L	F	5′ GAATCAAACACAACTAAACCTAAATTGGGTAATTTCCCACCACTG 3′
G3 F380/382L	R	5′CAGTGGTGGGAAATTACCCAATTTAGGTTTAGTTGTGTTTGATGATTC 3′
G3 F33W	F	5′ CATAAGAAGAGTTCTTTCCATACCATCTATGCAGCATGTCTGGGGC 3′
G3 F33W	R	5′ GCCCCAGACATGCTGCATAGATGGTATGGAAAGAACTCTTCTTATG 3′

**Table 2:** Primer sequences for real-time RT-PCR.

Target	Orientation	Sequence
hIFN-β	Forward	5′-GGAGGACGCCGCATTGAC-3′
	Reverse	5′-TGATAGACATTAGCCAGGAGGTTC-3′
hCCL5	Forward	5′-CCAGCAGTCGTCTTTGTCAC-3′
	Reverse	5′-CTCTGGGTTGGCACACACTT-3′
hCXCL10 (IP-10)	Forward	5′-TTCCTGCAAGCCAATTTTGTC-3′
	Reverse	5′-TCTTCTCACCCTTCTTTTTCATTGT-3′
hGAPDH	Forward	5′-GCAAATTCCATGGCACCGT-3′
	Reverse	5′-TCGCCCCACTTGATTTTGG-3′
hIFIT1 (ISG56)	Forward	5′-TACAGCAACCATGAGTACAA-3′
	Reverse	5′-TCAGGTGTTTCACATAGGC-3′
mIFN-β	Forward	5′-GAAAGGACGAACATTCGGAAAT-3′
	Reverse	5′-TCCGTCATCTCCATAGGGATCT-3′
mCCL5	Forward	5′-GCTGCTTTGCCTACCTCTCC-3′
	Reverse	5′-TCGAGTGACAAACACGACTGC-3′
mIFIT1 (ISG56)	Forward	5′-AGGGCTCTGCTACAAGCAACA-3′
	Reverse	5′-TGCCAATTCTTGCACATTGTC-3′
mIL-6	Forward	5′-TAGTCCTTCCTACCCCAATTTCC-3′
	Reverse	5′-TTGGTCCTTAGCCACTCCTTC-3′
mISG15	Forward	5′-GGTGTCCGTGACTAACTCCAT-3′
	Reverse	5′-TGGAAAGGGTAAGACCGTCCT-3′

## Data Availability

The original contributions presented in this study are included in the article/Supplementary Materials. Further inquiries can be directed to the corresponding author.

## Supplementary Materials

The following supporting information can be downloaded at: https://www.mdpi.com/article/10.3390/v18070719/s1, Figure S1: Role of TIA1 in SG formation and STING translocation; Figure S2: PERK activity does not affect STING translocation; Figure S3: Analysis of SG formation by G3BP1 mutants.

## References

[1] Kawai T., Akira S. (2006). Innate immune recognition of viral infection. Nat. Immunol..

[2] Kawai T., Akira S. (2010). The role of pattern-recognition receptors in innate immunity: Update on Toll-like receptors. Nat. Immunol..

[3] Chen Q., Sun L., Chen Z.J. (2016). Regulation and function of the cGAS-STING pathway of cytosolic DNA sensing. Nat. Immunol..

[4] Ishikawa H., Barber G.N. (2008). STING is an endoplasmic reticulum adaptor that facilitates innate immune signalling. Nature.

[5] Ishikawa H., Ma Z., Barber G.N. (2009). STING regulates intracellular DNA-mediated, type I interferon-dependent innate immunity. Nature.

[6] Sun L., Wu J., Du F., Chen X., Chen Z.J. (2013). Cyclic GMP-AMP synthase is a cytosolic DNA sensor that activates the type I interferon pathway. Science.

[7] Zhang Z., Zhang C. (2025). Regulation of cGAS-STING signalling and its diversity of cellular outcomes. Nat. Rev. Immunol..

[8] Zhong B., Yang Y., Li S., Wang Y.Y., Li Y., Diao F., Lei C., He X., Zhang L., Tien P. (2008). The adaptor protein MITA links virus-sensing receptors to IRF3 transcription factor activation. Immunity.

[9] Shang G., Zhang C., Chen Z.J., Bai X.C., Zhang X. (2019). Cryo-EM structures of STING reveal its mechanism of activation by cyclic GMP-AMP. Nature.

[10] Ablasser A., Goldeck M., Cavlar T., Deimling T., Witte G., Rohl I., Hopfner K.P., Ludwig J., Hornung V. (2013). cGAS produces a 2′-5′-linked cyclic dinucleotide second messenger that activates STING. Nature.

[11] Burdette D.L., Monroe K.M., Sotelo-Troha K., Iwig J.S., Eckert B., Hyodo M., Hayakawa Y., Vance R.E. (2011). STING is a direct innate immune sensor of cyclic di-GMP. Nature.

[12] Civril F., Deimling T., de Oliveira Mann C.C., Ablasser A., Moldt M., Witte G., Hornung V., Hopfner K.P. (2013). Structural mechanism of cytosolic DNA sensing by cGAS. Nature.

[13] Gao P., Ascano M., Wu Y., Barchet W., Gaffney B.L., Zillinger T., Serganov A.A., Liu Y., Jones R.A., Hartmann G. (2013). Cyclic [G(2′,5′)pA(3′,5′)p] is the metazoan second messenger produced by DNA-activated cyclic GMP-AMP synthase. Cell.

[14] Li X., Shu C., Yi G., Chaton C.T., Shelton C.L., Diao J., Zuo X., Kao C.C., Herr A.B., Li P. (2013). Cyclic GMP-AMP synthase is activated by double-stranded DNA-induced oligomerization. Immunity.

[15] Zhang C., Shang G., Gui X., Zhang X., Bai X.C., Chen Z.J. (2019). Structural basis of STING binding with and phosphorylation by TBK1. Nature.

[16] Zhao B., Du F., Xu P., Shu C., Sankaran B., Bell S.L., Liu M., Lei Y., Gao X., Fu X. (2019). A conserved PLPLRT/SD motif of STING mediates the recruitment and activation of TBK1. Nature.

[17] Yum S., Li M., Fang Y., Chen Z.J. (2021). TBK1 recruitment to STING activates both IRF3 and NF-kappaB that mediate immune defense against tumors and viral infections. Proc. Natl. Acad. Sci. USA.

[18] Kang J., Wu J., Liu Q., Wu X., Zhao Y., Ren J. (2022). Post-Translational Modifications of STING: A Potential Therapeutic Target. Front. Immunol..

[19] Dobbs N., Burnaevskiy N., Chen D., Gonugunta V.K., Alto N.M., Yan N. (2015). STING Activation by Translocation from the ER Is Associated with Infection and Autoinflammatory Disease. Cell Host Microbe.

[20] Mukai K., Konno H., Akiba T., Uemura T., Waguri S., Kobayashi T., Barber G.N., Arai H., Taguchi T. (2016). Activation of STING requires palmitoylation at the Golgi. Nat. Commun..

[21] Liu S., Cai X., Wu J., Cong Q., Chen X., Li T., Du F., Ren J., Wu Y.T., Grishin N.V. (2015). Phosphorylation of innate immune adaptor proteins MAVS, STING, and TRIF induces IRF3 activation. Science.

[22] Tanaka Y., Chen Z.J. (2012). STING specifies IRF3 phosphorylation by TBK1 in the cytosolic DNA signaling pathway. Sci. Signal..

[23] Wang C., Wang X., Veleeparambil M., Kessler P.M., Willard B., Chattopadhyay S., Sen G.C. (2020). EGFR-mediated tyrosine phosphorylation of STING determines its trafficking route and cellular innate immunity functions. EMBO J..

[24] Jeltema D., Abbott K., Yan N. (2023). STING trafficking as a new dimension of immune signaling. J. Exp. Med..

[25] He J., Zhang L. (2024). The journey of STING: Guiding immune signaling through membrane trafficking. Cytokine Growth Factor Rev..

[26] Huang T., Sun C., Du F., Chen Z.J. (2025). STING-induced noncanonical autophagy regulates endolysosomal homeostasis. Proc. Natl. Acad. Sci. USA.

[27] Gui X., Yang H., Li T., Tan X., Shi P., Li M., Du F., Chen Z.J. (2019). Autophagy induction via STING trafficking is a primordial function of the cGAS pathway. Nature.

[28] Liu B., Carlson R.J., Pires I.S., Gentili M., Feng E., Hellier Q., Schwartz M.A., Blainey P.C., Irvine D.J., Hacohen N. (2023). Human STING is a proton channel. Science.

[29] Lv B., Dion W.A., Yang H., Xun J., Kim D.H., Zhu B., Tan J.X. (2024). A TBK1-independent primordial function of STING in lysosomal biogenesis. Mol. Cell.

[30] Xun J., Zhang Z., Lv B., Lu D., Yang H., Shang G., Tan J.X. (2024). A conserved ion channel function of STING mediates noncanonical autophagy and cell death. EMBO Rep..

[31] Wu J., Dobbs N., Yang K., Yan N. (2020). Interferon-Independent Activities of Mammalian STING Mediate Antiviral Response and Tumor Immune Evasion. Immunity.

[32] Dunphy G., Flannery S.M., Almine J.F., Connolly D.J., Paulus C., Jonsson K.L., Jakobsen M.R., Nevels M.M., Bowie A.G., Unterholzner L. (2018). Non-canonical Activation of the DNA Sensing Adaptor STING by ATM and IFI16 Mediates NF-kappaB Signaling after Nuclear DNA Damage. Mol. Cell.

[33] Hou Y., Liang H., Rao E., Zheng W., Huang X., Deng L., Zhang Y., Yu X., Xu M., Mauceri H. (2018). Non-canonical NF-kappaB Antagonizes STING Sensor-Mediated DNA Sensing in Radiotherapy. Immunity.

[34] Zhang D., Liu Y., Zhu Y., Zhang Q., Guan H., Liu S., Chen S., Mei C., Chen C., Liao Z. (2022). A non-canonical cGAS-STING-PERK pathway facilitates the translational program critical for senescence and organ fibrosis. Nat. Cell Biol..

[35] Devale T., Katuri L., Mishra G., Acharya A., Manivannan P., Hibbard B.R., Malathi K. (2026). Cytosolic Immunostimulatory DNA Ligands and DNA Damage Activate the Integrated Stress Response, Stress Granule Formation, and Cytokine Production. Cells.

[36] Eom E., Kim J., Kim J., Kang S.J. (2026). STING is the scaffold protein for stress granule pre-condensation at the ER. Cell Death Differ..

[37] Pakos-Zebrucka K., Koryga I., Mnich K., Ljujic M., Samali A., Gorman A.M. (2016). The integrated stress response. EMBO Rep..

[38] Gale M., Tan S.L., Katze M.G. (2000). Translational control of viral gene expression in eukaryotes. Microbiol. Mol. Biol. Rev. MMBR.

[39] Kedersha N., Ivanov P., Anderson P. (2013). Stress granules and cell signaling: More than just a passing phase?. Trends Biochem. Sci..

[40] Sanders D.W., Kedersha N., Lee D.S.W., Strom A.R., Drake V., Riback J.A., Bracha D., Eeftens J.M., Iwanicki A., Wang A. (2020). Competing Protein-RNA Interaction Networks Control Multiphase Intracellular Organization. Cell.

[41] Yang P., Mathieu C., Kolaitis R.M., Zhang P., Messing J., Yurtsever U., Yang Z., Wu J., Li Y., Pan Q. (2020). G3BP1 Is a Tunable Switch that Triggers Phase Separation to Assemble Stress Granules. Cell.

[42] Guillen-Boixet J., Kopach A., Holehouse A.S., Wittmann S., Jahnel M., Schlussler R., Kim K., Trussina I., Wang J., Mateju D. (2020). RNA-Induced Conformational Switching and Clustering of G3BP Drive Stress Granule Assembly by Condensation. Cell.

[43] Manivannan P., Siddiqui M.A., Malathi K. (2020). RNase L Amplifies Interferon Signaling by Inducing Protein Kinase R-Mediated Antiviral Stress Granules. J. Virol..

[44] Onomoto K., Yoneyama M., Fung G., Kato H., Fujita T. (2014). Antiviral innate immunity and stress granule responses. Trends Immunol..

[45] Yoneyama M., Jogi M., Onomoto K. (2016). Regulation of antiviral innate immune signaling by stress-induced RNA granules. J. Biochem..

[46] White J.P., Lloyd R.E. (2012). Regulation of stress granules in virus systems. Trends Microbiol..

[47] McCormick C., Khaperskyy D.A. (2017). Translation inhibition and stress granules in the antiviral immune response. Nat. Rev. Immunol..

[48] Liu Z.S., Cai H., Xue W., Wang M., Xia T., Li W.J., Xing J.Q., Zhao M., Huang Y.J., Chen S. (2019). G3BP1 promotes DNA binding and activation of cGAS. Nat. Immunol..

[49] Zhao M., Xia T., Xing J.Q., Yin L.H., Li X.W., Pan J., Liu J.Y., Sun L.M., Wang M., Li T. (2022). The stress granule protein G3BP1 promotes pre-condensation of cGAS to allow rapid responses to DNA. EMBO Rep..

[50] Aulas A., Fay M.M., Szaflarski W., Kedersha N., Anderson P., Ivanov P. (2017). Methods to Classify Cytoplasmic Foci as Mammalian Stress Granules. J. Vis. Exp..

[51] Li X., Chen X., Zheng L., Chen M., Zhang Y., Zhu R., Chen J., Gu J., Yin Q., Jiang H. (2023). Non-canonical STING-PERK pathway dependent epigenetic regulation of vascular endothelial dysfunction via integrating IRF3 and NF-kappaB in inflammatory response. Acta Pharm. Sin. B.

[52] Chin A.C. (2020). PERK-STING Signaling Drives Neuroinflammation in Traumatic Brain Injury. J. Neurosci. Off. J. Soc. Neurosci..

[53] Jeremiah N., Neven B., Gentili M., Callebaut I., Maschalidi S., Stolzenberg M.C., Goudin N., Fremond M.L., Nitschke P., Molina T.J. (2014). Inherited STING-activating mutation underlies a familial inflammatory syndrome with lupus-like manifestations. J. Clin. Investig..

[54] Wang C., Nan J., Holvey-Bates E., Chen X., Wightman S., Latif M.B., Zhao J., Li X., Sen G.C., Stark G.R. (2023). STAT2 hinders STING intracellular trafficking and reshapes its activation in response to DNA damage. Proc. Natl. Acad. Sci. USA.

[55] Du M., Chen Z.J. (2018). DNA-induced liquid phase condensation of cGAS activates innate immune signaling. Science.

[56] Yang B., Pei J., Lu C., Wang Y., Shen M., Qin X., Huang Y., Yang X., Zhao X., Ma S. (2023). RNF144A promotes antiviral responses by modulating STING ubiquitination. EMBO Rep..

[57] Hu M.M., Yang Q., Xie X.Q., Liao C.Y., Lin H., Liu T.T., Yin L., Shu H.B. (2016). Sumoylation Promotes the Stability of the DNA Sensor cGAS and the Adaptor STING to Regulate the Kinetics of Response to DNA Virus. Immunity.

[58] Wu J., Yan N. (2019). STIM1 moonlights as an anchor for STING. Nat. Immunol..

[59] Srikanth S., Woo J.S., Wu B., El-Sherbiny Y.M., Leung J., Chupradit K., Rice L., Seo G.J., Calmettes G., Ramakrishna C. (2019). The Ca(2+) sensor STIM1 regulates the type I interferon response by retaining the signaling adaptor STING at the endoplasmic reticulum. Nat. Immunol..

[60] Luo W.W., Li S., Li C., Lian H., Yang Q., Zhong B., Shu H.B. (2016). iRhom2 is essential for innate immunity to DNA viruses by mediating trafficking and stability of the adaptor STING. Nat. Immunol..

[61] Zhang B.C., Nandakumar R., Reinert L.S., Huang J., Laustsen A., Gao Z.L., Sun C.L., Jensen S.B., Troldborg A., Assil S. (2020). STEEP mediates STING ER exit and activation of signaling. Nat. Immunol..

[62] Chu T.T., Tu X., Yang K., Wu J., Repa J.J., Yan N. (2021). Tonic prime-boost of STING signalling mediates Niemann-Pick disease type C. Nature.

[63] Horan K.A., Hansen K., Jakobsen M.R., Holm C.K., Soby S., Unterholzner L., Thompson M., West J.A., Iversen M.B., Rasmussen S.B. (2013). Proteasomal degradation of herpes simplex virus capsids in macrophages releases DNA to the cytosol for recognition by DNA sensors. J. Immunol..

[64] Krawczyk E., Kangas C., He B. (2023). HSV Replication: Triggering and Repressing STING Functionality. Viruses.

[65] Deng L., Xu Z., Li F., Zhao J., Jian Z., Deng H., Lai S., Sun X., Geng Y., Zhu L. (2022). Insights on the cGAS-STING Signaling Pathway During Herpesvirus Infections. Front. Immunol..

[66] Dauber B., Saffran H.A., Smiley J.R. (2019). The herpes simplex virus host shutoff (vhs) RNase limits accumulation of double stranded RNA in infected cells: Evidence for accelerated decay of duplex RNA. PLoS Pathog..

[67] Zhu H., Zheng C. (2020). The Race between Host Antiviral Innate Immunity and the Immune Evasion Strategies of Herpes Simplex Virus 1. Microbiol. Mol. Biol. Rev. MMBR.

