# Supplementary figures and images for: Multiple Roles of G3BP1 in Regulating STING-Dependent Interferon and Cytokine Induction by Cytosolic dsDNA and HSV-1 Infection
Source: Viruses. 2026 Jun 30;18(7):719. doi: 10.3390/v18070719 (PMC13431489; doi:10.3390/v18070719)

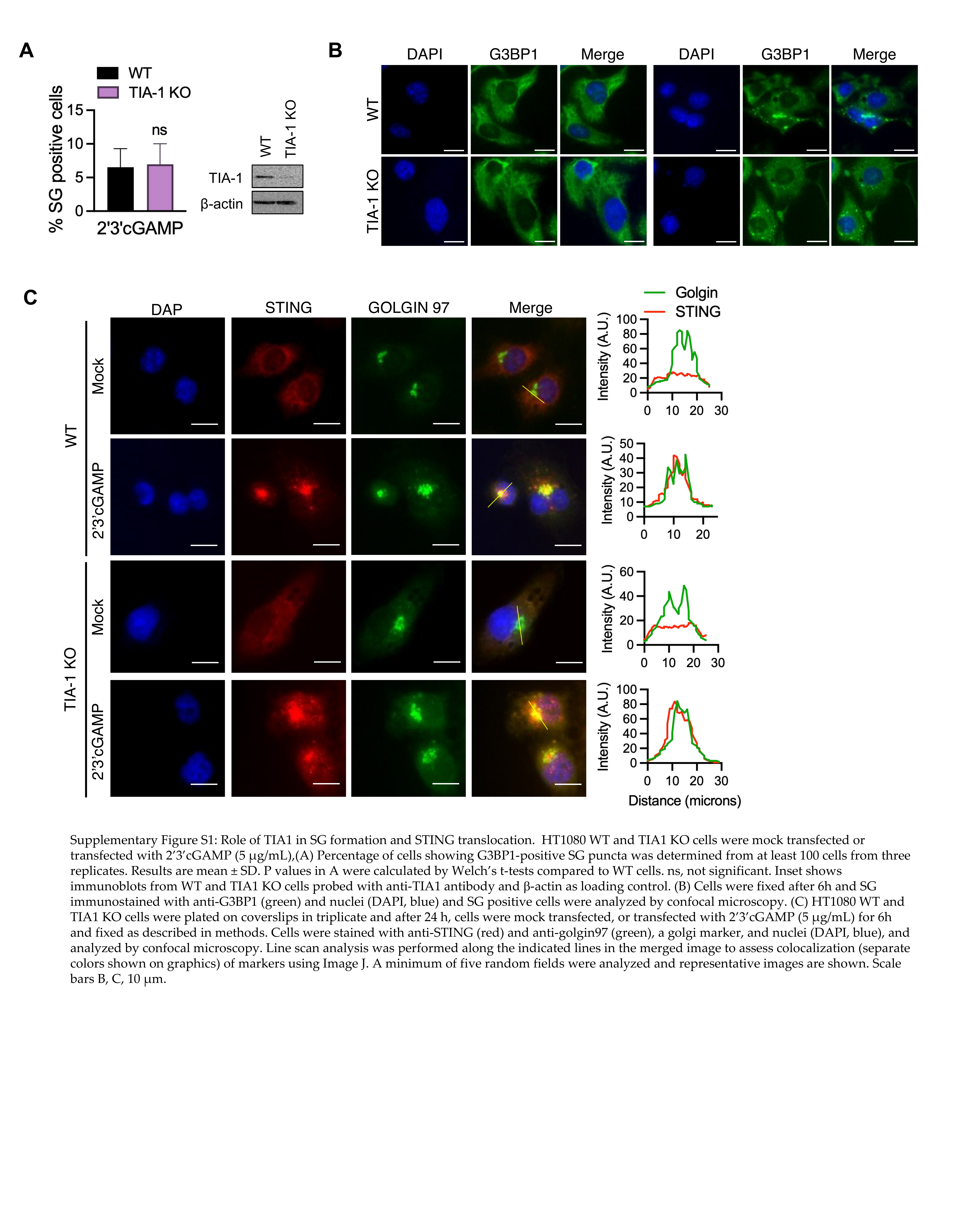

Supplement: Supplementary file 1 [file viruses-18-00719-s001.zip › Supplementary figure S1 image.png]

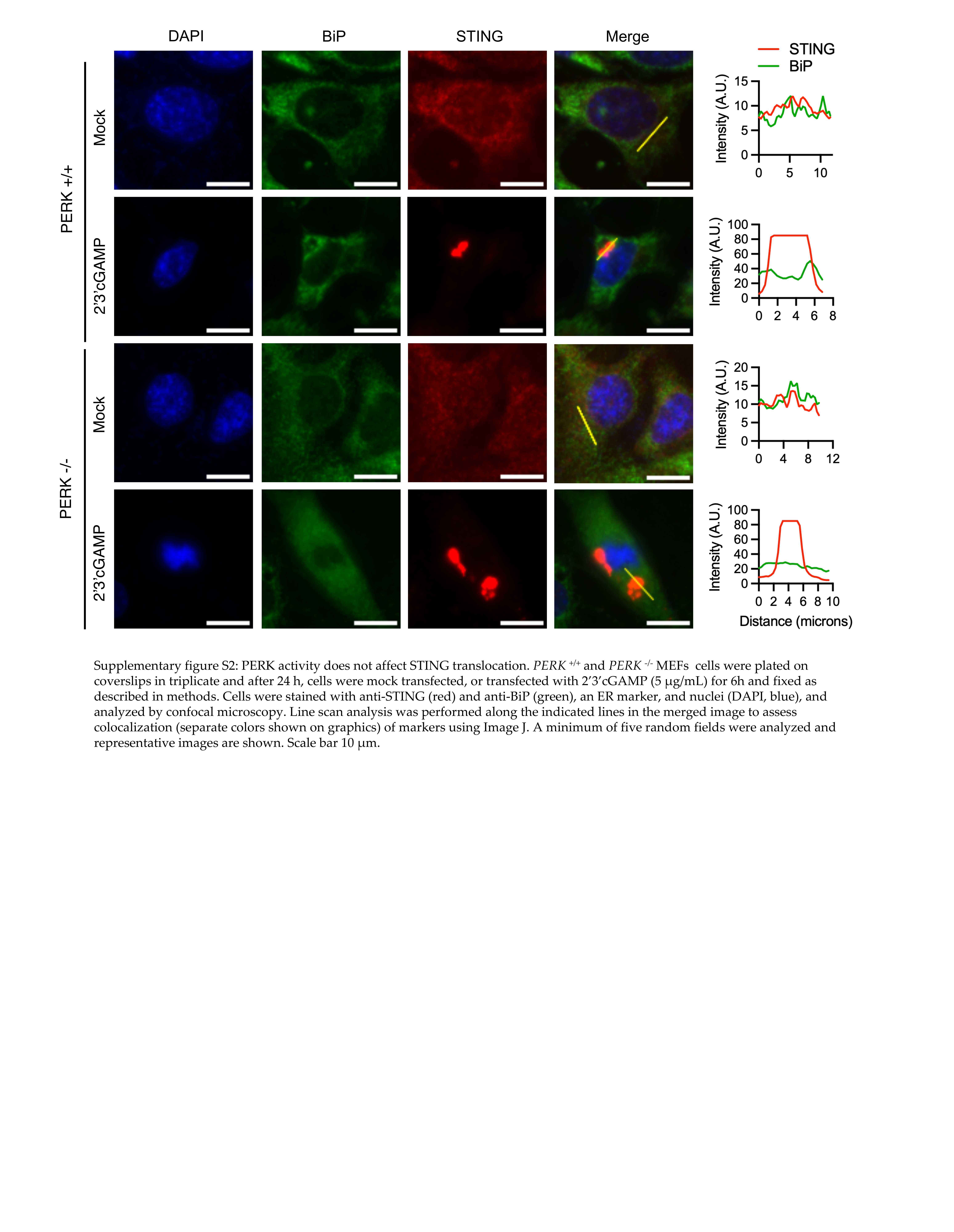

Supplement: Supplementary file 1 [file viruses-18-00719-s001.zip › Supplementary figure S2 with legend_fixed.png]

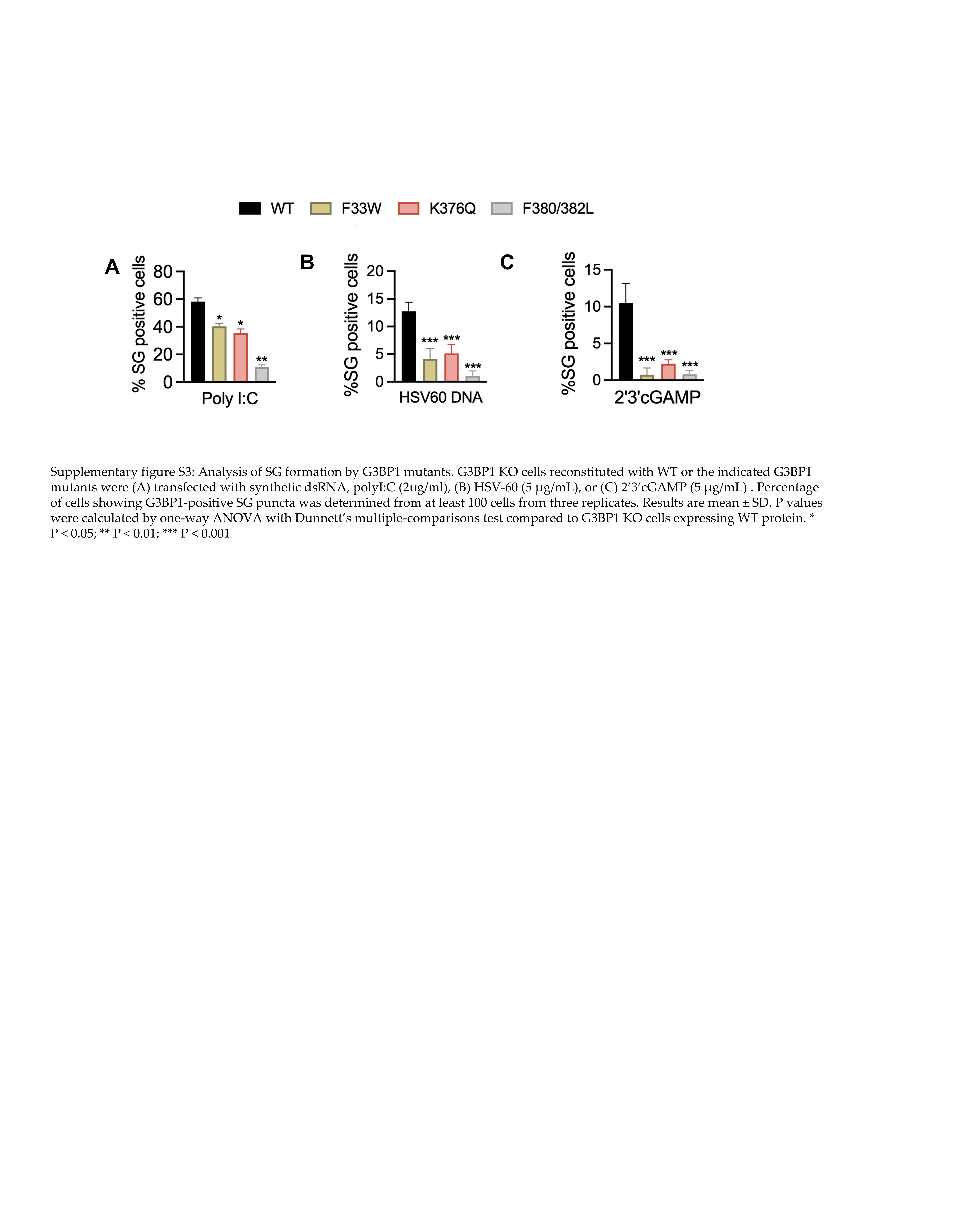

Supplement: Supplementary file 1 [file viruses-18-00719-s001.zip › Supplementary figure S3 image.png]
